# Combinatorial action of Grainyhead, Extradenticle and Notch in regulating Hox mediated apoptosis in *Drosophila* larval CNS

**DOI:** 10.1371/journal.pgen.1007043

**Published:** 2017-10-12

**Authors:** Risha Khandelwal, Rashmi Sipani, Sriivatsan Govinda Rajan, Raviranjan Kumar, Rohit Joshi

**Affiliations:** 1 Laboratory of Drosophila Neural Development, Centre for DNA Fingerprinting and Diagnostics (CDFD), Tuljaguda Complex, Nampally, Hyderabad, India; 2 Graduate Studies, Manipal University, Manipal, India; Duke-NUS Medical School, SINGAPORE

## Abstract

Hox mediated neuroblast apoptosis is a prevalent way to pattern larval central nervous system (CNS) by different Hox genes, but the mechanism of this apoptosis is not understood. Our studies with Abdominal-A (Abd-A) mediated larval neuroblast (pNB) apoptosis suggests that AbdA, its cofactor Extradenticle (Exd), a helix-loop-helix transcription factor Grainyhead (Grh), and Notch signaling transcriptionally contribute to expression of RHG family of apoptotic genes. We find that Grh, AbdA, and Exd function together at multiple motifs on the apoptotic enhancer. *In vivo* mutagenesis of these motifs suggest that they are important for the maintenance of the activity of the enhancer rather than its initiation. We also find that Exd function is independent of its known partner homothorax in this apoptosis. We extend some of our findings to Deformed expressing region of sub-esophageal ganglia where pNBs undergo a similar Hox dependent apoptosis. We propose a mechanism where common players like Exd-Grh-Notch work with different Hox genes through region specific enhancers to pattern respective segments of larval central nervous system.

## Author summary

Specification of the head to tail axis of the developing central nervous system is carried out by Hox genes. Hox mediated programmed cell death of the neural progenitor cells plays an important role in specification of this axis, but the molecular mechanism of this phenomenon is not well understood. We have studied this phenomenon in abdominal and subesophageal regions of larval central nervous system of Drosophila. We find that different Hox genes use a combination of common players (Extradenticle, Grainyhead and Notch) but employ region specific enhancers to cause progenitor cell death in different segments of developing central nervous system.

## Introduction

Apoptosis is used to eliminate defective and/or dispensable cell types during development of an organism. Removal of excess cells from the developing tissue is critical for its final size, shape and functionality in the whole organismal context [1–3]. The central nervous system (CNS) also relies heavily on apoptosis for its patterning and development [4–7].

Equally important and one of the earliest steps in development of CNS is specification of anterior posterior axis (AP axis). AP axis specification is carried out by Hox genes [8–11] and their TALE homeodomain containing cofactors; Extradenticle (Exd) and Homothorax (Hth) in *Drosophila* [12,13]; and Pbx and Meis in vertebrates [14]. Hox genes pattern CNS by regulating proliferation, differentiation and apoptosis of different cell types [15–22] [23,24]. Their role in developmental apoptosis of CNS has been reported earlier, both in *Drosophila* [16–18] as well as in vertebrates [21,22], but the molecular mechanism of the cell death in neural stem cells as well as their progeny are not known.

In *Drosophila*, Hox mediated apoptosis of neural stem cells (neuroblast-NB) and their progeny happens both in embryonic and post-embryonic (larval) stages of development [16–18,23–30]. This apoptosis is mediated through activation of RHG family of genes (*reaper*, *hid*, *grim* and *sickle*) [31–34], but the precise molecular mechanism tying a particular Hox gene to death of a specific cell type is not known. In larval stages, Hox mediated NB apoptosis has been reported for Labial (Lab), Deformed (Dfd), Sex combs reduced (Scr) and Abdominal-A (AbdA) expressing regions of CNS [16,23,24]. AbdA mediated larval NB (postembryonic NB-pNB) apoptosis is so far the best characterized of all [16,35], yet the precise molecular details of the same are lacking.

The segments A3-A7 of embryonic ventral nerve cord has 60 NBs each (30 per hemi-segment). Following an embryonic wave of AbdA mediated NB apoptosis [17], majority of NBs undergo cell death, leaving behind only 3 NBs per hemisegment. Following embryonic phase of apoptosis, abdominal NBs stop expressing AbdA and enter quiescence by the end of embryogenesis. All the 3 NBs have specific locations and developmental potential and are designated as NB5-2 (Ventromedial-Vm), NB5-3 (Ventrolateral-Vl) and NB3-5 (Dorsolateral-Dl) in embryonic stages, and Lineage-6, Lineage-5 and Lineage-9 in larval stages respectively [36,37]. These 6 NBs (3 per hemisegment) exit quiescence in early third instar larval (L3) stage (66–72 hours after egg laying-AEL) and divide for different durations, following which an asynchronous increase in AbdA expression in these cells causes their apoptosis and removal from CNS over a course of next 48 hours [16,35]. The pNBs mutant for either *abdA*, *or grainyhead* (*grh*-a basic helix-loop-helix (bHLH) transcription factor) or RHG genes [as seen in genomic deletion-*Df(3L)H99*] escape apoptosis, underlying their individual importance in cell death [16,35]. The mechanistic details of how Grh and AbdA mediate pNB apoptosis through RHG genes is not known.

Similarly, in subesophageal ganglia (SEG) of larval CNS (which expresses Dfd, Scr and Antennapedia), 36 NBs (18 segmental pairs) are reported in second instar larval (L2) stage. Out of these 36 pNBs (18 pairs), 10 pNBs (5 pairs) are found in Dfd expressing region of SEG [24], here on referred to as Dfd-SEG. Four out of these 10 pNBs undergo Dfd mediated apoptosis as larva progresses from L2 to L3 stage (illustrated later in a Figure and detailed in S1 Text) [24]. The molecular mechanism of this apoptosis and role of Grh in this phenomenon is yet to be investigated.

A genomic deletion analysis had identified a 22kb region referred to as *NBRR* (*NeuroBlast Regulatory Region*), which contains NB specific enhancer for apoptotic genes [38]. A 5 Kb subfragment (*enh-1*) of *NBRR*, has been suggested to be the apoptotic enhancer required for embryonic NBs. However, it is not clear whether the same enhancer functions to cause larval NB apoptosis [25]. This study also suggests that pulse of AbdA expression responsible for larval NB apoptosis, is initiated in response to activation of Notch signaling in these cells [25].

In this report, we have investigated the molecular basis of Hox mediated larval pNB apoptosis. We analyzed 22 Kb *NBRR* and have narrowed down the larval abdominal apoptotic enhancer to a 1Kb region of the genome. Our experiments suggest that AbdA, Exd, Grh and Notch transcriptionally contribute to regulation of RHG genes, and Exd has a Hth independent role in this apoptosis. *In vitro* experiments suggest that AbdA and Grh physically interact with each other, and Grh-AbdA-Exd assemble a tetrameric complex with DNA on some of the binding motifs in the apoptotic enhancer. *In vivo* mutagenesis of all the motifs suggest, that they are important not for the initiation but for the maintenance of the enhancer activity, and consequently expression of RHG genes. Our analysis of the enhancer mutant for Su(H) binding sites reveal that Notch signaling also has a direct input in the maintenance of the enhancer activity and hence RHG genes in abdominal pNBs.

Subsequently we show that Dfd mediated pNB apoptosis in SEG use same players (Hox-Exd-Grh-Notch) but employ a different enhancer located outside *NBRR*.

Taken together, this study describes a common mechanism of RHG gene regulation in pNBs undergoing Hox dependent apoptosis. Wherein combination of Exd-Grh-Notch are employed by specific Hox genes, to carry out apoptosis in different regions of developing CNS through separate spatial enhancers.

## Results

### Analysis of 22Kb NBRR

**Fig 1:**
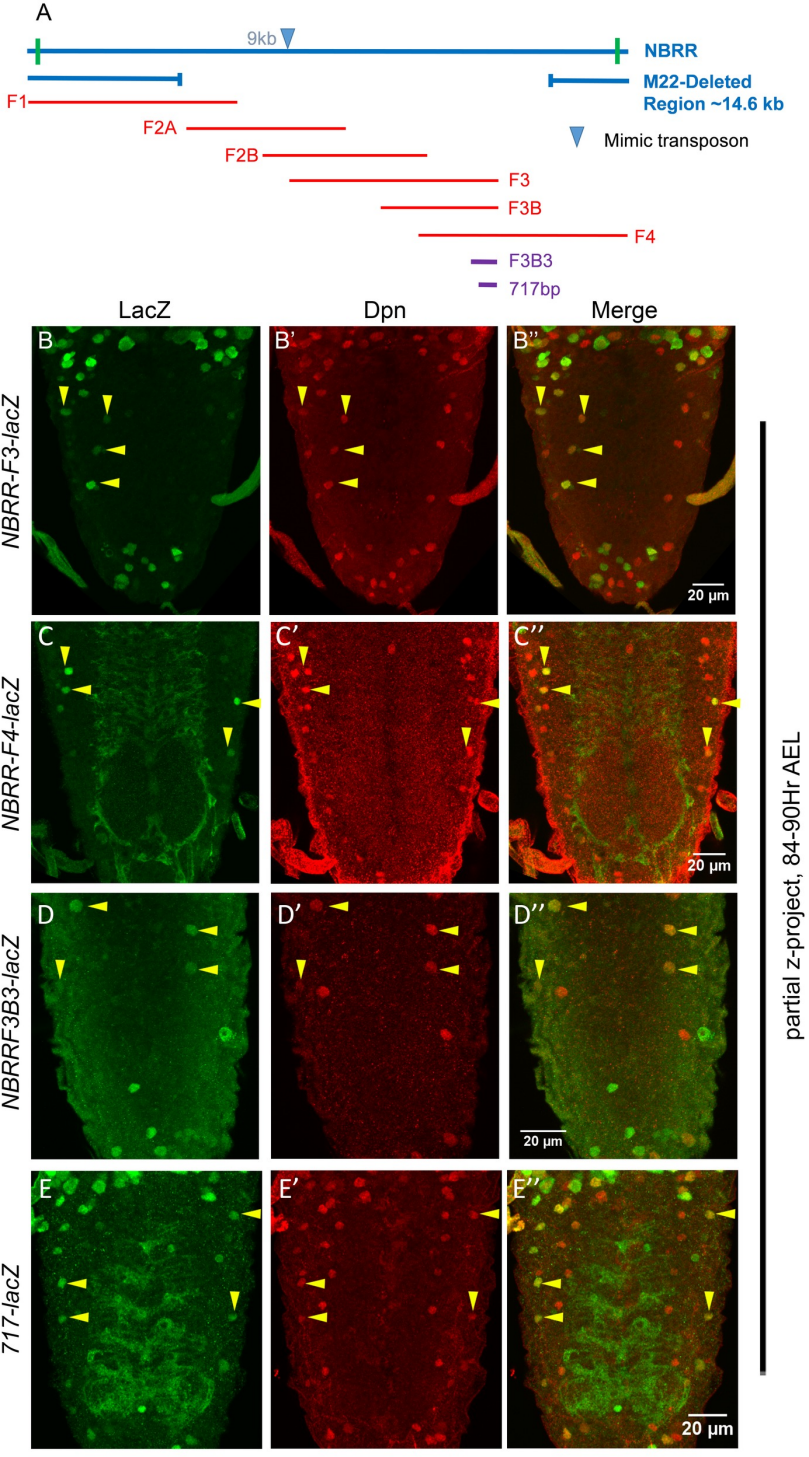
Enhancer for abdominal NB apoptosis lies in *NBRR F3* and *F4* overlap. (A) Schematic showing 22kb *NBRR* region with approximate extents of *M22* deletion and overlapping genomic fragments used for making *enhancer-lacZ* lines. MiMIC transposon used to generate the *M22* deletion is inserted at approximate 9kb from 5’ end of *NBRR*, indicated by blue arrowhead. (B-E) Show mid L3 larval VNCs for *NBRR F3*, *F4*, *F3B3* and *717 enhancer-lacZ* lines. Yellow arrowheads indicate pNBs.

23 Kb genomic region (including 22Kb *NBRR* and additional 500bps on either side; Fig 1A) was divided into 5 over lapping fragments (Fig 1A). LacZ reporter lines were generated for these fragments and analyzed for their expression in larval CNS (Fig 1, S1 Table and S1 Fig). Since AbdA pulse doesn’t come on simultaneously in all the pNBs, therefore these cells die asynchronously. NBs start dying from early L3 stage and over a period of next 48 hrs different pNBs activate AbdA at different times and undergo apoptosis with majority of death happening between mid to late L3 stages. Owing to this, we chose to look at the larval ventral nerve cords (VNC) in time range of 84–90 hrs AEL. At this time, we expected majority of abdominal pNBs to be lacZ^+^. The reporter lines for *NBRR* fragments *F1* (8 Kb), *F2A* (6 Kb) and *F2B* (6 Kb) failed to show any lacZ expression in abdominal pNBs (S1A–S1C Fig). However, *NBRR fragment-3* (8 Kb) and *fragment-4* (8 Kb) reporter lines (here on referred to as *F3-lacZ* and *F4-lacZ*) expressed in pNBs of abdominal CNS (Fig 1B and 1C). This indicated that the apoptotic enhancer lies in 3 Kb overlapping region of *F3* and *F4* fragments. This was further confirmed by analyzing the expression of the reporter line made from last 4.5 Kb region of *F3* (referred to as *F3B-lacZ*, S1D Fig). Subsequent reporter lines were made by subfragmenting 3Kb overlapping region, of which a 1Kb reporter lacZ line (referred to as *F3B3-lacZ*) recapitulated larval pNB expression in abdominal pNBs at 84-90hrs AEL (Fig 1D).

In order to isolate the smallest modular enhancer, a 717bp subfragment was further selected from 1 Kb based on its sequence conservation across multiple *Drosophila* species, chromatin accessibility and the presence of multiple transcription factor (TF) binding sites, as assessed by UCSC genome browser (S2 Fig) [39]. The transgenic line for 717bp subfragment (referred to as *717-lacZ*, Fig 1E) was generated by site specific insertion [40]. We found that this reporter line expressed in pNB at 84–90 hrs AEL, but the expression of the reporter was limited to Vl pNBs at late L3 stage. Our analysis suggests that this was a consequence of the insertion of the construct at the specific site (attP40-25C6), since insertion of 1Kb *F3B3-lacZ* at the same site also restricted its expression to Vl pNBs (S1E Fig). Owing to this, even though *717-lacZ* expressed in pNBs (Fig 1E) and exhibited other features of genuine apoptotic enhancer (S3 Fig), specific insertion site seems to have altered its activity. Therefore, majority of following experiments were done with 1Kb *F3B3-lacZ*, and 717 bp enhancer was mainly considered for *in vivo* binding site mutant analysis.

In order to assess the temporal regulation of these genomic sub-fragments, we analyzed the expression of 8kb *F3*, 1kb *F3B3* and *717-lacZ* at early L3 stage (66-72hrs AEL), which was a few hours prior to initiation of pNB apoptosis (S3 Fig). We found all the reporter lines expressed weakly in abdominal pNBs and their expression was limited only to a few abdominal pNBs. However, as larvae progress to mid L3 stage, the expression is extended into more pNBs (Fig 1B–1E), suggesting that these *enhancer-lacZ* lines reflect the temporal control of RHG gene expression.

In order to genetically isolate the enhancer, we also generated a 14.6 Kb genomic deletion called *M22* (detailed in S1 Text) which deletes the entire *F3* fragments (Fig 1A and S4D Fig). We observed that heteroallelic combination of *M22/MM3* (*MM3* is a 54 Kb deletion used earlier to isolate *NBRR* [38]) showed ectopic NBs in the abdominal region of CNS (161.9+/-12.7, n = 20 VNCs, S4B and S4C Fig). The number of ectopic pNBs were comparable to those observed in *MM3/MM3* deletion (167.6+/-10.8, for n = 12 VNCs), suggesting that 14.6Kb *M22* deletion uncovers the enhancer for abdominal pNB apoptosis.

The reporter line expression and *M22* deletion analysis strongly suggested that enhancer for abdominal pNB lies within 1Kb *F3B3* region of *NBRR*.

### Apoptotic enhancer sustain its expression till late L3 stage

Since, we could capture lacZ^+^ abdominal pNBs with reasonable frequency, this indicated to us that lacZ expression in these cells was not immediately followed by cell death. We also observed that intensity of lacZ expression in pNBs in early L3 stage was weak and became stronger in mid L3 stage (Compare S3 Fig and Fig 1B–1E). This suggested that lacZ reporter expression (and RHG genes) comes on and then sustain itself, till these cells undergo apoptosis. This is congruent to what is reported earlier in case of *grim* deletion where NB death is delayed till late L3 stages, when *rpr* finally executes the cell death [38].

**Fig 2:**
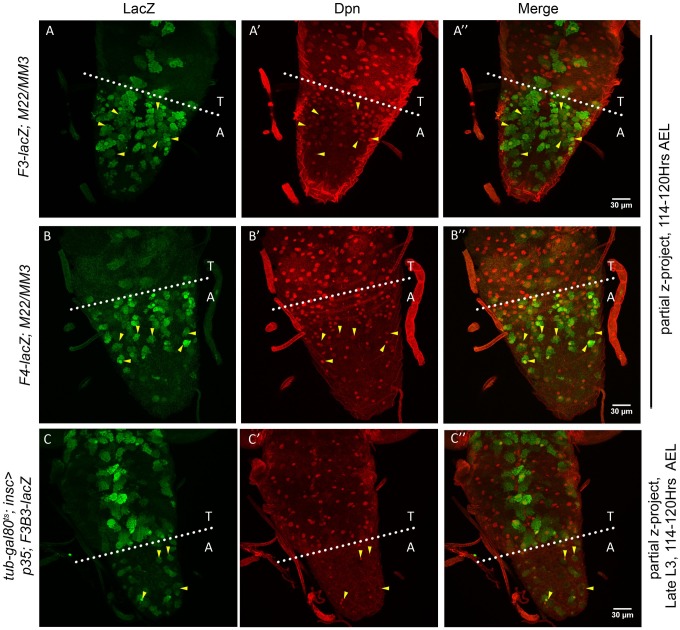
*Enhancer-lacZ* expression sustains till late L3 stage in abdominal NBs. (A-B) Show expression of *NBRR F3* and *F4 enhancer-lacZ* lines in abdominal NBs resulting from block of apoptosis in *M22/MM3* transheterzygotic deletion combination. (C) Show that pNBs resulting from blocking of death by expression of p35 in L1 stage (t-shift as shown in S8A Fig) express *F3B3-lacZ*. Yellow arrowheads indicate pNBs.

Considering these observations, we expected that the apoptotic enhancer should be capable of maintaining the expression of the lacZ reporter (and RHG genes) in abdominal pNBs even till late L3 stage of development. To this end, we tested different reporter lines (*F3*, *F4*, *F3B*, *F3B3*, Fig 2A–2C, and *717-lacZ* shown with later results) for their sustained expression in pNBs destined for apoptosis. This was achieved by testing the expression of lacZ lines in cell death blocked background by either using genetic deletions (*for NBRR*) or by expression of apoptosis blocker p35.

We observed that both *F3* and *F4-lacZ* lines expressed in abdominal pNBs as late as 114–120 hrs AEL (Fig 2A and 2B) in *M22/MM3* transheterozygotic background.

In order to conclusively confirm that larval NBs which undergo AbdA mediated apoptosis express the reporter line, and this expression sustain till late L3 stage we used *tub-GAL80*^*ts*^*; insc-*GAL4 driven *UAS-p35* expression. This was used to temporally block NB apoptosis specifically from first instar larval stage (L1) (t-shift as shown in S8A Fig). We found that reporter lines *F3B3-lacZ* (Fig 2C) and *717-lacZ* (shown with later results) expressed in the surviving pNBs as late as 114–120 hrs AEL.

Collectively these observations suggest that apoptotic enhancer expression once initiated in a pNB is maintained till it undergoes death.

### Grh and AbdA transcriptionally regulate apoptotic enhancer

Grh has been reported to be expressed in CNS from embryonic stage 11. Its expression in larvae is limited to NBs and is excluded from neurons. Since CNS specific *grh* mutants show a block in abdominal pNB apoptosis [35,41,42], we decided to investigate its role in AbdA mediated pNB apoptosis.

**Fig 3:**
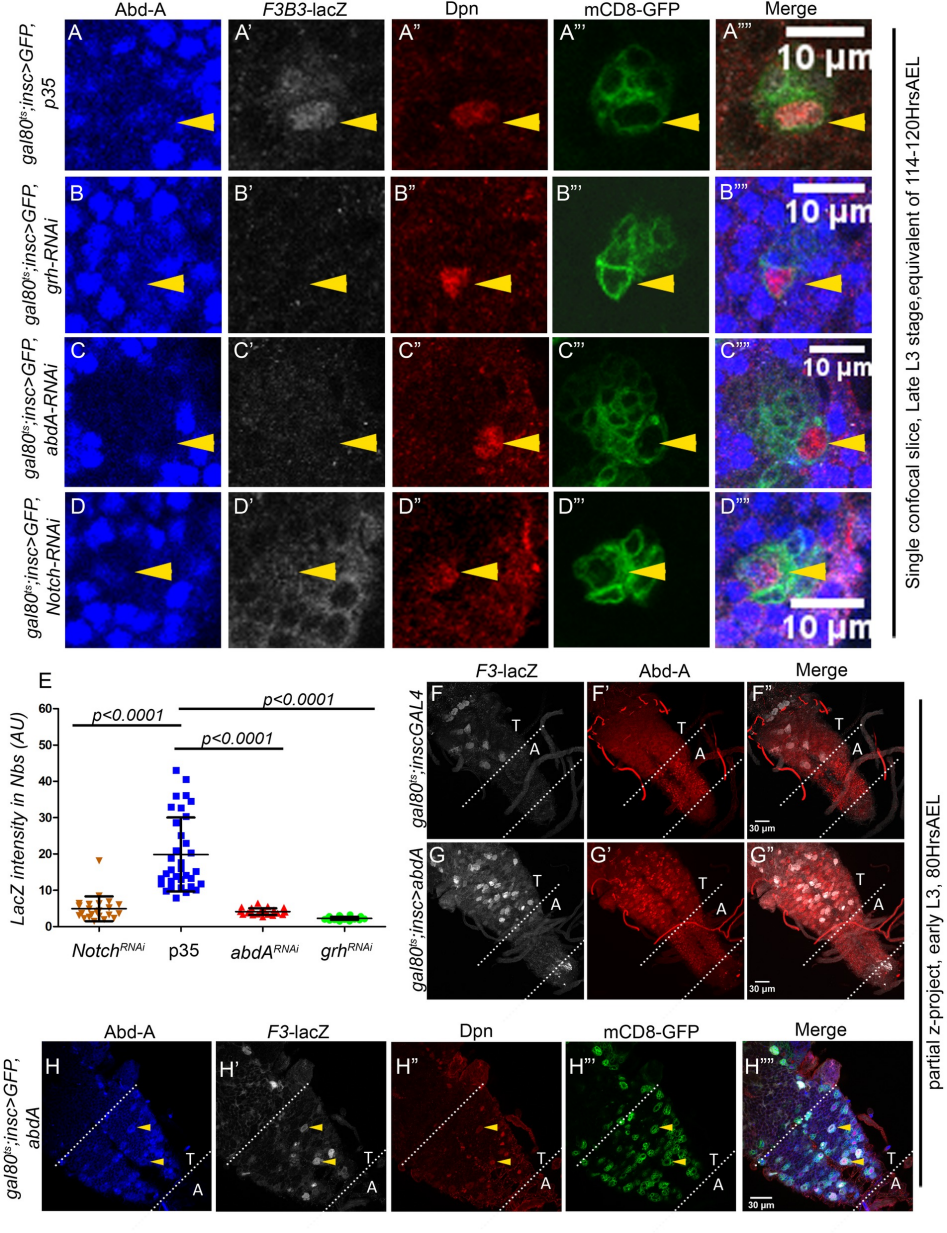
Grh, Abd-A and Notch transcriptionally regulate apoptotic enhancer. (A-D) Shows *F3B3-lacZ* levels in abdominal pNBs in response to *grh* (B), *abd-A* (C) and *Notch* (D) knockdown compared with pNB blocked for apoptosis by p35 expression (A). (E) Shows the plot of LacZ intensities quantitated and compared from VNCs where abdominal pNBs are blocked from undergoing death by expression of p35 versus VNCs with *grh*, *abd-A* and *Notch* knockdown. (F-G) Comparison of control and Abd-A over expressed larval VNCs with *F3-lacZ*. (F) Shows basal level of *F3-lacZ* expression in thoracic NBs. (G) Shows induction of *F3-lacZ* (white channel) in additional cells in response to ectopic expression of AbdA in thoracic segments of CNS. The dotted lines in these panels enclose abdominal segments of larval VNC which normally express Abd-A. (H) Shows enlarged thoracic region of VNC shown in panel-G. Induction of *F3-lacZ* and Abd-A in Dpn marked pNB in thoracic segments is shown. The dotted lines in these panels enclose thoracic segments of larval VNC. Yellow arrowheads indicate pNBs. Abdominal and Thoracic segments are indicated as “A” and “T”. Average values are shown as central lines in error bars. Error bars indicate standard deviation.

To this end, we used RNA interference (RNAi) to knock down *grh*, *abdA* and *Notch* (discussed in later section) in pNBs and score for their effect on 1Kb *F3B3-lacZ* reporter line expression. *tub-gal80*^*ts*^ was used to temporally induce the knockdown from late embryonic stage and larvae were dissected in late L3 stage (114–120 hrs AEL; t-shift as shown in S8B Fig). The effect of these knockdowns on *F3B3-lacZ* in abdominal pNBs was quantitated and compared to lacZ levels from pNBs blocked for apoptosis by expression of p35. In order to maintain uniformity of comparison, pNBs of ventromedial (Vm) lineages have been quantitated across all genotypes (Fig 3E). We found *F3B3-lacZ* expression to be consistently downregulated in pNBs when *grh* (n = 26 pNBs, Average intensity = 2.3+/-0.4), *abdA* (n = 23 pNBs, Average intensity = 4.1+/-0.9) and *Notch* (n = 23 pNBs, Average intensity = 4.9+/- 3.4) were knocked down (Fig 3B-3B””, 3C–3C”” and 3D–3D””), as compared to pNBs expressing p35 (n = 34 pNBs, Average intensity = 19.9+/-10.2) (Fig 3A–3A””). The trend and significance of the data was unchanged across multiple experimental sets. One such set has been presented in the Fig 3. To rule out sample variations, Dpn staining for the NBs across different genotypes was quantified and found to be comparable (S5I Fig).

Ectopic expression of AbdA in thoracic pNBs is known to cause their apoptosis. Therefore, we expected that *enhancer-lacZ* expression should also get induced in these cells in response to ectopic AbdA (Grh is already present in these cells). Ectopic expression of AbdA was induced from early L3 stage and larvae were dissected 7 and 12 hrs later in early and mid L3 stage for *F3* and *F4-lacZ* respectively (t-shift as shown in S8F Fig). We observed that *F3-lacZ* expressed in very few thoracic pNBs (Fig 3F) in control VNCs, whereas the ectopic expression of AbdA induced *F3-lacZ* in many thoracic pNBs (Fig 3G). Consistent with this observation, *F4-lacZ* expression was also ectopically induced (S5A and S5B Fig). We also found that induction of lacZ happens primarily in pNBs as indicated by co-staining for AbdA, Dpn and lacZ (Fig 3H). The lacZ expression seen in some of the progeny is a consequence of these cells inheriting lacZ from their progenitors. Similarly, we observed 1Kb *F3B3* and *717-lacZ* also get induced in response to AbdA over expression (S5E–S5H and S5G and S5H Fig). Since these smaller subfragments were more promiscuous in their expression in thoracic region, induction in response to AbdA was scored by increase in intensity of lacZ expression in addition to ectopic expression in thoracic NBs. Ectopic expression of lacZ could also be detected in central brain as well (S5F and S5H Fig).

The inducible expression of *F3*, *F4*, *F3B3 and 717-lacZ* further suggested that apoptotic enhancer is responsive to ectopic induction of AbdA. Further, the knockdown suggest that AbdA and Grh transcriptionally regulate RHG genes in pNBs through 1Kb *F3B3* enhancer.

### Grh is important for pNB apoptosis in Dfd-SEG region

**Fig 4:**
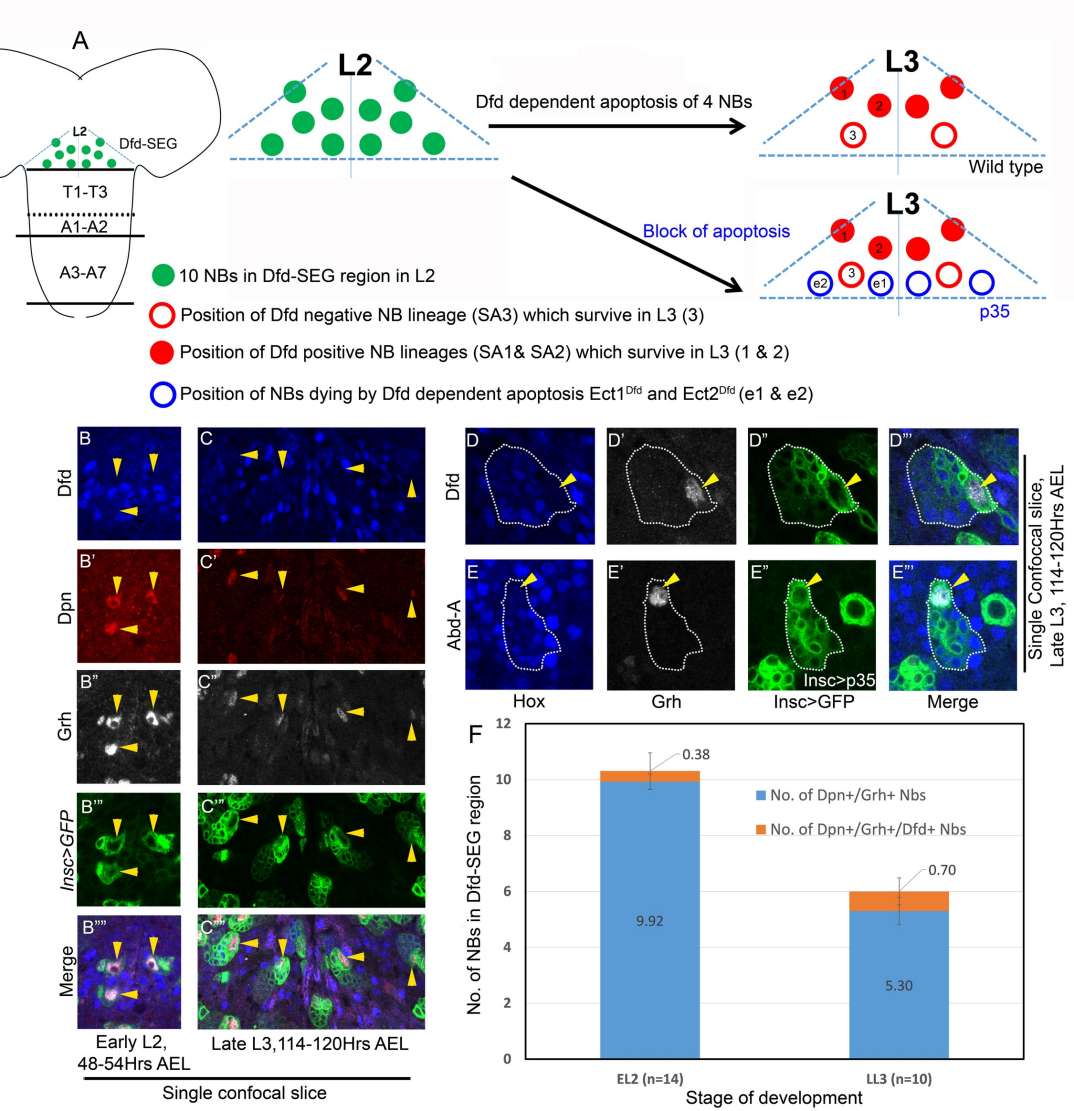
Hox-Grh expression code in pNBs and progeny is same for Dfd-SEG and abdominal segments. (A) Schematic of L2 VNC showing the extent of Dfd-SEG region as seen by Dfd staining *in vivo*. Ten pNBs in L2 stage is shown as green filled circles. Approximate location of six pNBs surviving in L3 stage are shown as four filled red circles (showing SA1 and SA2 lineages indicated as 1 and 2) and two hollow red circles (representing SA3 lineage indicated as 3). Approximate location of four ectopic lineages when death is blocked by p35 expression from L1 are shown by hollow blue circles (*ect1*^*Dfd*^ and *ect2*^*Dfd*^ are indicated as e1 and e2) (B-C) Show that pNB in Dfd-SEG region of early L2 and late L3 larval VNCs express Grh. (D-E) Shows that Hox-Grh code of the pNB (Hox^-^/Grh^+^) and progeny (Hox^+^/Grh^-^) is same in abdominal and Dfd-SEG region. GFP marked lineage is enclosed by a dotted line. Biggest cell in the entire lineage is pNB which is Grh^+^, smaller cells are progeny neurons which are Hox^+^ and Grh^-^. pNB death in abdominal segments is blocked by *gal80*^*ts*^*; inscGAL4* driven UAS-p35 expression specifically in larval stages. (F) Plot shows that all the pNB marked by Dpn in EL2 (10) and LL3 (6) are Grh^+^. Small “n” indicates number of larval VNCs counted. All larval VNCs expressed *inscGAL4* driven *UAS-mCD8-GFP* from embryonic stages. Yellow arrowheads indicate pNBs. Average values are shown in middle of bars. Error bars indicate standard deviation.

Out of ten pNBs in Dfd-SEG in L2 stage, four undergo Dfd dependent apoptosis as animal progresses to L3 stage of development (Fig 4A) [24]. In order to investigate molecular basis of this apoptosis (Fig 4A), we first checked whether Grh was expressed in pNBs found in L2 stage in Dfd-SEG region. These pNB lineages were identified by their location in Dfd stained region of SEG. The pNBs were marked by Dpn and the whole lineage was marked by *inscGAL4* driven *UAS-mCD8-GFP* expression (Fig 4B and 4C). We consistently found 10 pNBs (9.92+/-0.27, for n = 13 L2 VNCs) which were Dpn^+^/Grh^+^ (Fig 4F) in early L2 stage (48–54 hrs AEL). Only few of these early L2 pNBs showed very low but detectable levels of Dfd (0.38+/- 0.65 for n = 13 L2 VNCs) shown as Dpn^+^/Grh^+^/Dfd^+^ (in Fig 4F). By late L3 stage (114–120 hrs AEL) only 6 pNBs and associated lineages were observed. We found that pNBs in these lineages always expressed Grh (6.0+-/-0, for more than 20 L3 VNCs) (Fig 4C and 4F) and showed very low or no expression of Dfd. As in L2 stage, pNBs were consistently Grh^+^/Dpn^+^ (5.3+/-0.48, for n = L3 VNCs) (Fig 4C” and 4F) and Dfd negative (Fig 4C and 4F). On a closer observation of the lineages in both abdominal and Dfd-SEG segments of VNCs, we found that Hox-Grh code of pNBs and their progeny was same: pNBs were always Grh^+^/Hox^-^, progeny were always Grh^-^/Hox^+^ (checked for more than 20 L3 VNCs) (Fig 4D–4D’ and 4E–4E’).

This implied that apoptosis of pNBs in Dfd-SEG may also be Grh dependent, and is possibly triggered by change in Hox^-^/Grh^+^ state of pNB to Hox^+^/Grh^+^ state. This prompted us to test the functional role of Grh in this apoptosis by knocking down its expression using genetic mutant combination and RNAi.

**Fig 5:**
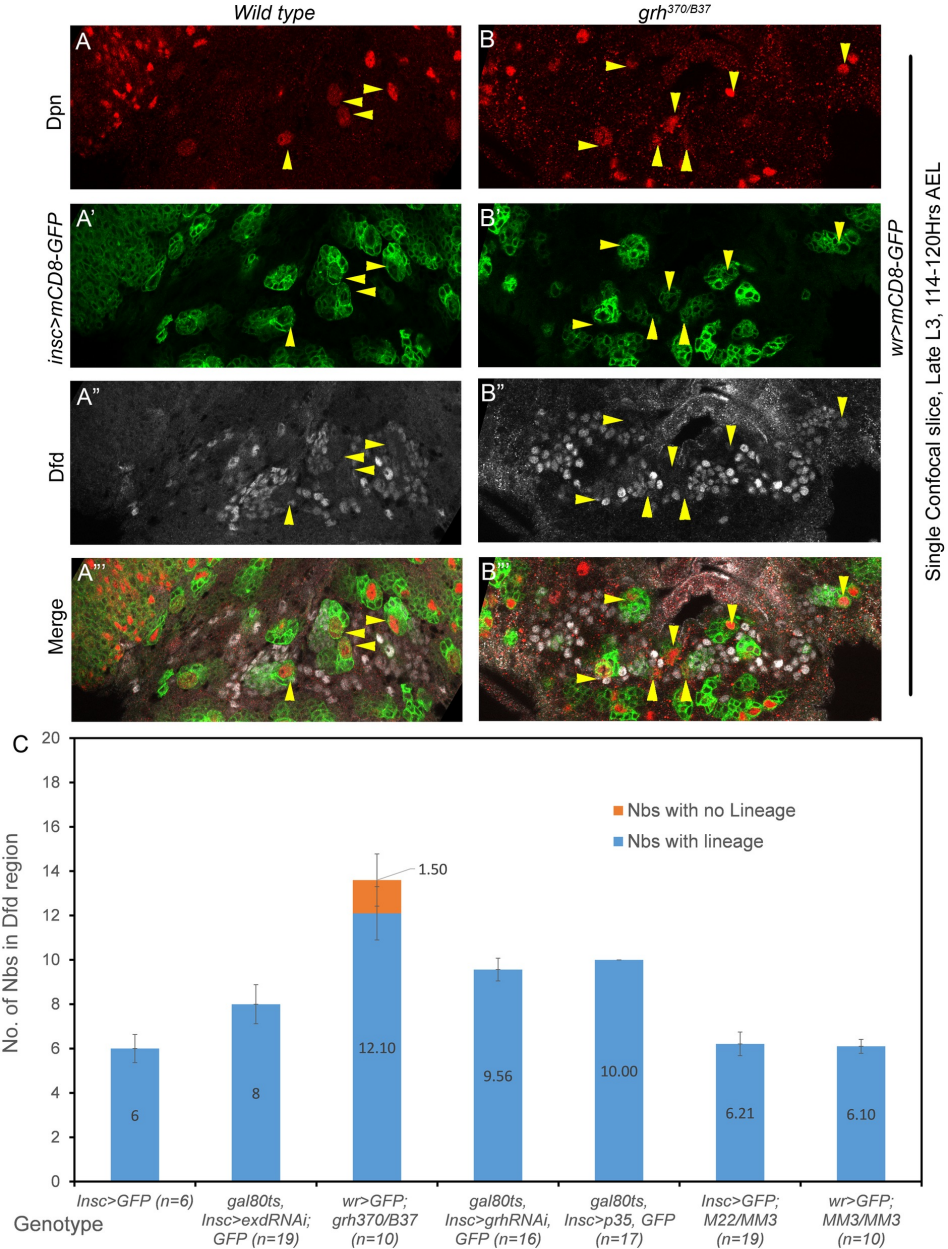
Analysis of NBs surviving in Dfd region of SEG. (A) Single confocal section of wild type VNC showing only four out of six pNB lineages normally surviving in Dfd-SEG. (B) Show seven pNB lineages found in single confocal section of *grh* mutant VNC (*grh*^*370/B37*^; *wrGAL4> UAS-mCD8-GFP*). 7 NB lineages are visualized in this section, suggesting that Grh is important for pNB apoptosis in Dfd-SEG region. (C) Plot showing number of surviving NBs counted in Dfd-SEG region in various genotypes. “n” indicates the number of late L3 VNCs counted in each case. Majority of the pNBs in *grh*^*370/B37*^ mutant combination were associated with lineages; (12.1+/-1.2, for n = 10 L3 VNCs) only a small fraction were without any associated lineages (1.5+/-1.2, for n = 10 L3 VNC). Yellow arrowheads indicate pNBs. Average values are shown in middle of the bars. Error bars indicate standard deviation.

To this end, we counted and compared the number of pNBs in Dfd-SEG region of *grh*^*370/B37*^ (CNS specific null allelic combination for *grh*) with wild type controls. In wild type late L3 stage VNC (*insc>mCD8-GFP*, 114–120 hrs AEL, Fig 5A–5A”‘), we counted 6 pNBs (6.0+/-0.6, for more than 20 L3 VNCs, Fig 5C, bar-1 of the graph) as expected [24]. For the ease of representation Fig 5A shows a single confocal section with 4 of these 6 pNBs (marked by yellow arrowheads, Fig 5A–5A”‘). However, in case of *grh* mutants (*grh*^*370/B37*^; *wr>mCD8-GFP*, in late L3 stage Fig 5B–5B”‘) an average 13 pNBs could be detected in Dfd-SEG region (13.6+/-0.5, for n = 10 L3 VNCs, Fig 5C, bar-3 of the graph). Representation show a single confocal section of the mutant VNC where seven out of total 14 NBs found in Dfd-SEG region are shown (Fig 5B–5B”‘). Remaining ectopic pNBs were in other confocal planes and hence are not visible here. Since the number of NBs in case of *grh* mutant was more than ten we expected some of NBs to be embryonic in origin.

In order to conclusively establish the contribution of Grh in larval NB apoptosis we induced RNAi mediated *grh* knockdown specifically from late embryonic stages (by this time embryonic NB death has already taken place) and dissected the larvae in late L3 stage at 114–120 hrs AEL (t-shift as shown in S8B Fig). Ectopic pNBs in Dfd-SEG were identified and counted based on their position and Dfd staining. The detailed method for identification of ectopic pNBs in Dfd-SEG are given in S1 Text.

We found 4 ectopic pNB lineages (total of 10 pNB lineages) in late L3 VNCs (9.56+/-0.51, for n = 15 L3 VNCs) (Fig 5C, bar-4 of the graph). These numbers were in agreement with the fact that there are 10 pNBs reported in Dfd-SEG of wild type CNS in L2 stage, as well as when anti-apoptotic gene *p35* was specifically expressed from late embryonic stage (Fig 5C, bar-5 of the graph) or early L1 stage (10+/-0.0, for 7 VNCs).

These results show that similar to its role in abdominal segments, Grh also plays an important role in pNB apoptosis in Dfd-SEG region.

### Enhancer for pNB apoptosis in Dfd-SEG lies outside 22Kb *NBRR*

In order to identify the genomic location of the enhancer responsible for apoptosis of 4 pNBs in Dfd-SEG, we counted the number of ectopic pNBs in this region in late L3 stage for various deletion combinations. We could recover only 6 pNBs in Dfd-SEG region in *M22/MM3* (Fig 5C, bar-6 of the graph), *MM3/MM3* (Fig 5C, bar-7 of the graph). This implied that enhancer responsible for activation of apoptosis of pNBs in Dfd-SEG is different from abdominal apoptotic enhancer and lies outside 22Kb *NBRR* and 54Kb genomic region deleted in *MM3* allele.

### Role of Notch in pNB apoptosis is independent of Grh

**Fig 6:**
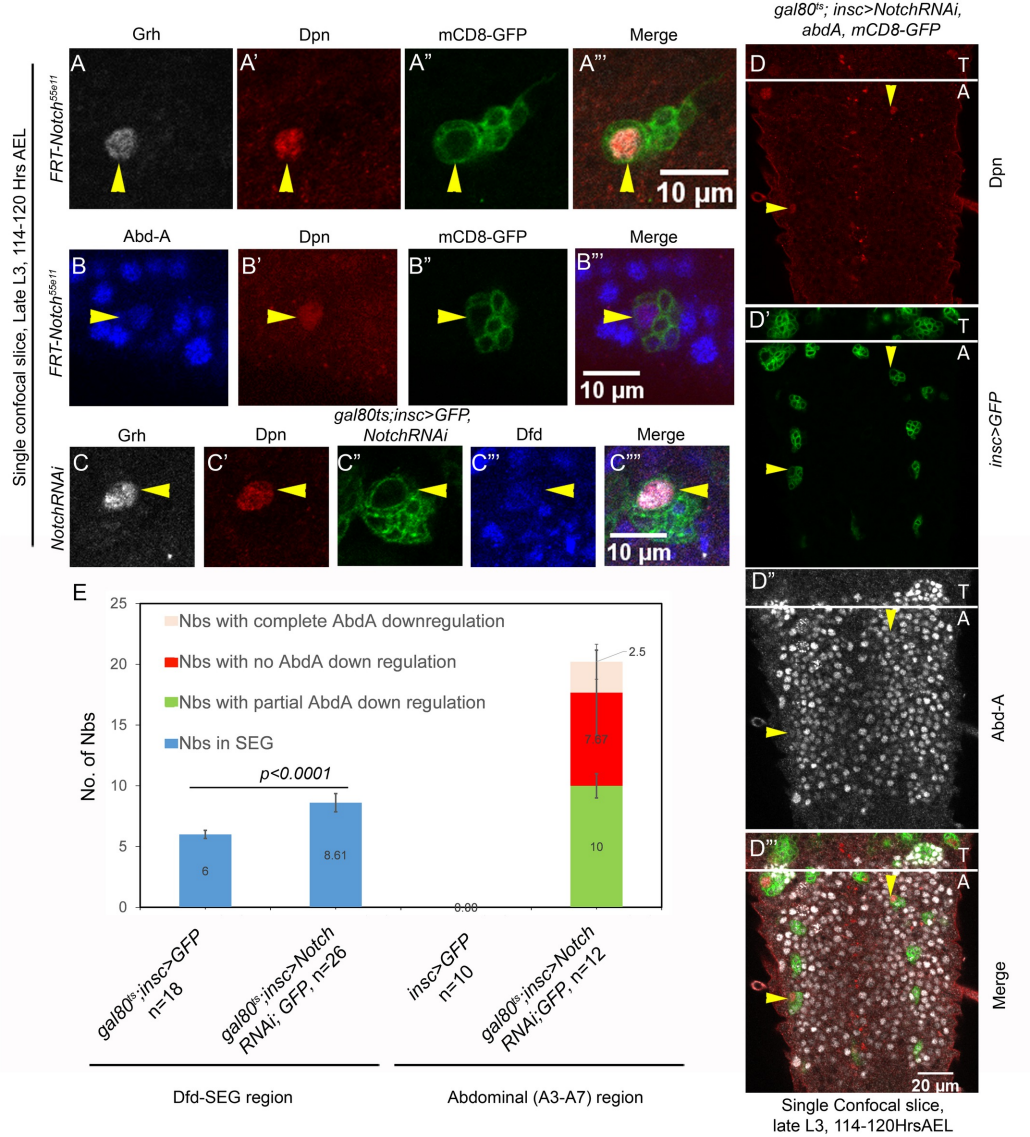
Role of Notch in pNB apoptosis is independent of Grh. (A-B) Show abdominal pNB MARCM clone for Notch mutation (*N*^*55e11*^). Grh expression is found to be normal (panel-A) and Abd-A is found to be expressed (panel-B) in pNBs. (C) Shows that Grh expression is unaffected in ectopic pNBs obtained in Dfd-SEG region in response to Notch knockdown by RNA interference. (D) Simultaneous knockdown of Notch (by RNA interference) and over expression of Abd-A in abdominal pNBs block their apoptosis. (E) Plot showing a comparison of the total number of pNBs in Dfd-SEG and abdominal region of larval VNCs in wild type and Notch knockdown by RNA interference. “n” indicate the number of VNCs counted for each genotype. Average values are shown in bars. Error bars are standard deviations.

Notch signaling is often utilized to make decisions in multiple developmental contexts [43–46]. More recently, it has been suggested to play a role in abdominal pNB apoptosis, where it was implicated in activating the pulse of AbdA triggering pNB apoptosis [25]. We first checked whether Notch has a lineage autonomous function in pNBs of abdominal segments by making Notch loss of function MARCM clones (*N*^*55e11*^). We recovered two kind of clones in abdominal region of CNS; the first class of clones were recovered in A3-A7 segments of CNS where the AbdA mediated abdominal pNB apoptosis is mainly reported (discussed below). A second class of clones were recovered in A1-A2 segments where no AbdA mediated apoptosis occurs (detailed in discussion). The clones recovered in A3-A7 segments had a surviving pNB at 114–120 hrs AEL indicating that pNB had failed to undergo apoptosis (Fig 6A and 6B). These clones were small and showed no consistent and significant downregulation of AbdA in the pNBs (compared to the levels of AbdA in progeny of the same lineage, Fig 6B). Therefore, we checked for the levels of Grh in these cells and found them to be unaffected in surviving pNBs (Fig 6A). Out of 23 clones examined across 10 VNCs, only 3 clones showed complete AbdA downregulation, 15 clones showed partial downregulation, and 5 clones showed no downregulation of AbdA. We also employed RNAi to verify these observations. Notch knockdown was initiated from late embryonic stage and its effect was examined in late L3 stage (t-shift as shown in S8B Fig). Three types of ectopic NBs were recovered; the first two type of NBs showed AbdA levels comparable or less than neuronal progeny of the lineage, these were designated as NBs with no or partial AbdA downregulation. The third type of NBs showed no AbdA expression (categorized as NBs showing complete downregulation). We report on an average 20 pNBs (20.0+/-2.7, n = 12 VNCs) surviving per VNC, of which only 2 pNBs (2.5+/-1.44, n = 12 VNCs) could show us complete downregulation, while out of remaining 17, 10 pNB (10+/-2.92, n = 12 VNCs) showed partial and 7 pNBs (7.67+/-3.5, n = 12 VNCs) showed no AbdA downregulation (Fig 6E). Thus, both RNAi and mutant data for Notch demonstrate that Notch probably does not induce AbdA expression in pNB of A3-A7 segment. Moreover, if Notch was indeed capable of inducing AbdA expression in pNBs, in that case overexpression of NICD (Notch Intracellular domain) in abdominal pNBs should induce AbdA and hence *F3-lacZ* in majority of these cells. We overexpressed NICD from mid L2 stage, and larvae were dissected after 14hrs at 30°C (approximately in early L3 stage, 70Hrs AEL, t-shift as shown in S8G Fig). However, we did not see ectopic induction of *F3-lacZ* in response to NICD overexpression in majority of the abdominal pNBs (S5L Fig).

These results suggested to us that Notch signaling may not be the trigger for AbdA induction in pNBs of A3-A7 segments but instead may have a direct role in pNB apoptosis. In order to validate this, we induced Notch knockdown and AbdA over expression simultaneously from early L1 stage (*gal80*^*ts*^*; inscGAL4>UAS-Notch-RNAi*, *UAS-abdA*, *UAS-mCD8-GFP*) and dissected larvae at late L3 stage of development (t-shift as shown in S8A Fig). If Notch signaling was working only through AbdA activation, over expression of AbdA in Notch knockdown background should have caused the apoptosis of abdominal pNBs. On the contrary, in our experiments, we found that Notch knockdown blocked apoptosis of abdominal pNBs even when AbdA was over expressed in these cells (16.8+/-3.2 surviving pNBs, in 11 VNCs, Fig 6D). Even though AbdA was expressed in surviving abdominal pNBs in late L3 stage (Fig 6D”), we wanted to ensure that sufficient levels of AbdA were expressed in pNBs in earlier stages, for this we analyzed VNCs in mid L3 stages as well. We found sufficient levels of AbdA were expressed in abdominal pNBs in mid L3 stage (S6A” and S6B” Fig). These observation and downregulation of apoptotic *enhancer-lacZ* in response to RNAi mediated knockdown of Notch (Fig 3D–3D””), further reinstate our proposition of a direct role of Notch in abdominal pNB apoptosis.

We also observed that the number of thoracic pNBs found in these VNCs were comparatively less in late L3 stages, which suggested that AbdA was able to cause apoptosis in thoracic region despite Notch knockdown in these cells. Noticeably, some of the surviving pNBs in thoracic segment expressed AbdA (S6D Fig). We think these cell types are refractory to AbdA mediated cell death. This possibly points towards a segment specific role of Notch in pNB apoptosis.

Next, we checked for the role of Notch signaling in pNB apoptosis in Dfd-SEG. For this RNAi mediated knockdown for Notch was induced from late embryonic stage and its effect was assessed in late L3 stage (t-shift as shown in S8B Fig). We could consistently recover 2–3 ectopic pNBs (8.61+/- 0.75, for n = 26 L3 VNCs) in Dfd-SEG as against 6 pNB seen in control larvae (Fig 6C and 6E). A closer observation of the ectopic pNBs show that they have normal levels of Grh (Fig 6C) and also expressed low levels of Dfd (Fig 6C). A comparative quantitation of pNBs recovered for both abdominal segments and Dfd-SEG region, upon Notch knockdown is shown in Fig 6E.

These results indicate a role of Notch signaling along with Hox and Grh in mediating pNB apoptosis in both abdominal and Dfd-SEG regions. In both cases, we found that Notch signaling does not impact the levels of Grh. Our results with abdominal pNBs further suggests that Notch knockdown is epistatic to AbdA overexpression in pNBs. Therefore, we believe that Notch does not regulate Hox expression and instead plays a direct role in pNB apoptosis.

### Exd but not Hth plays an important role in pNB apoptosis

**Fig 7:**
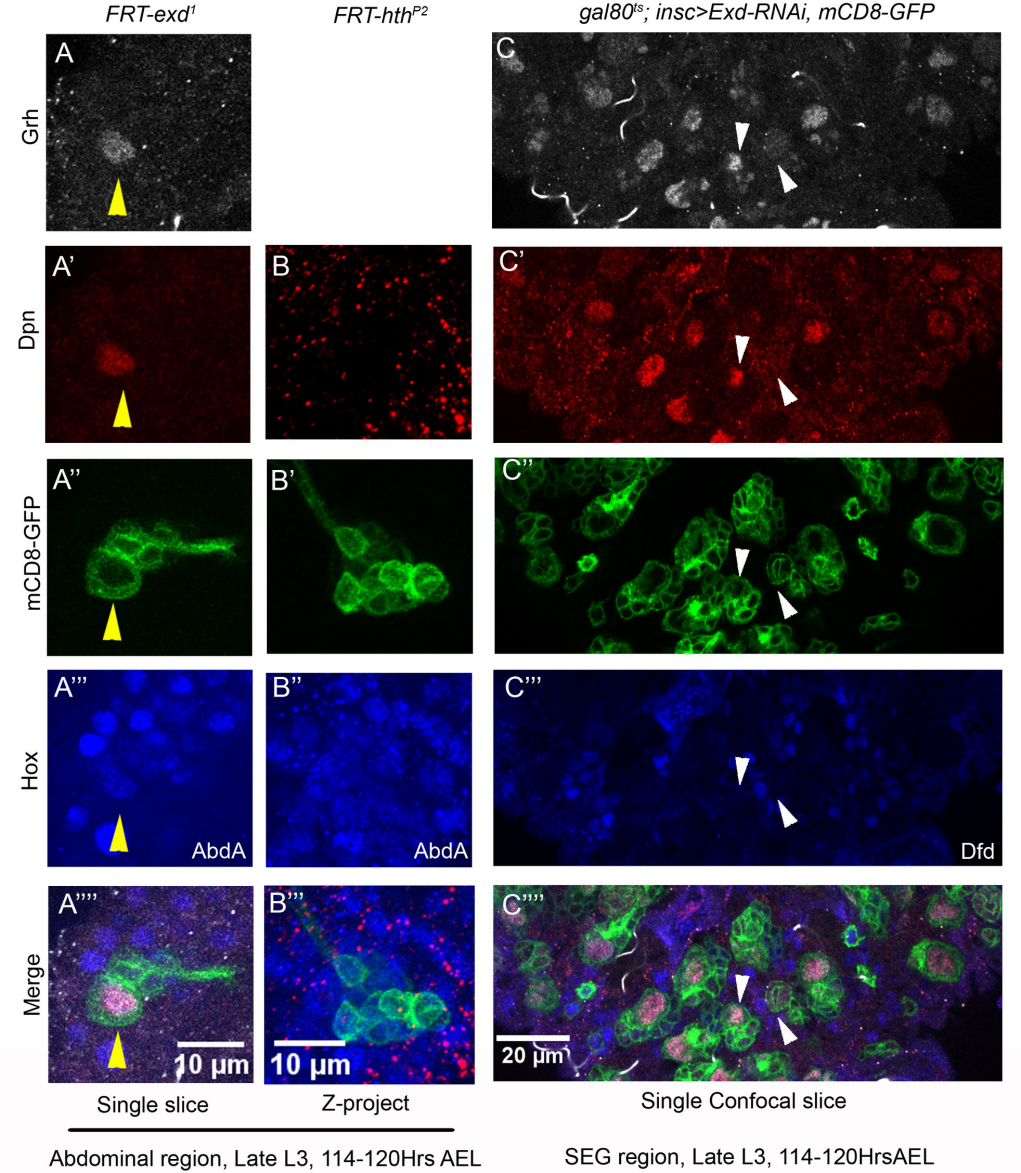
Exd but not Hth plays an important role in abdominal pNB apoptosis. (A-B) Show abdominal MARCM clone for Exd (*exd*^*1*^) and Hth (*hth*^*P2*^) mutations. pNB is seen in *exd*^*1*^ mutant clone marked by Dpn (A’) but *hth*^*P2*^ clone doesn’t show any surviving pNB (B). (C) Knockdown of Exd using RNA interference in Dfd-SEG region results in ectopic pNBs. Yellow and white arrowheads indicate ectopic NBs.

Hox genes have been known to function with two other TALE-HD containing transcription factors, Hth and Exd. We tested the role of Exd in pNB apoptosis in abdominal region by making MARCM clones. We recovered NB containing *exd* mutant clones in abdominal region (n = 16 clones in A3-A7 segment of 13 larval VNCs, Fig 7A–7A””). These pNB also showed a normal expression of Grh (n = 7 clones scored in 6 VNCs) and AbdA (Fig 7A). Exd is known to function with Hth, which helps in its nuclear localization [47]. Surprisingly, we found that *hth*^*P2*^ mutant clones in abdominal region did not block pNB apoptosis (Fig 7B–7B””). We noticed that clones were marked with GFP (Fig 7B’), suggesting that pNBs divided normally following the exit from quiescence but then underwent apoptosis (n = 23 clones scored in 10 VNCs). Since *hth*^*P2*^ is a strong hypomorph, we tested 6 *hth* RNAi lines. Even when RNAi mediated knockdown was induced from early embryonic stages for *hth* gene, we could not recover any ectopic pNBs in abdominal region (t-shift as shown in S8E Fig). Though we could see a visible down regulation of Hth expression in thoracic pNB lineages, suggesting that these lines were capable of inducing potent knockdown (S9 Fig).

These results led us to investigate a similar role for Exd and Hth in Dfd-SEG region as well. None of the *exd-RNAi* lines tested could give us ectopic pNBs in abdominal region. However, knockdown of *exd* from early embryonic stages (t-shift as shown in S8E Fig) resulted in approximately 2 ectopic pNBs in Dfd-SEG region in late L3 stage VNCs (1.75+/-0.61 for 19 VNCs) (Fig 7C). The ectopic NBs obtained were at the characteristic location where *ect1*^*Dfd*^ ectopic pNB lineage is normally observed and these lineages were Dfd^+^ as well. Similarly, we tested the role of Hth by 6 RNAi lines (as mentioned earlier) by inducing knockdown from early embryonic stages but we could not find any significant difference between control VNCs and knockdown VNCs in late L3 stage (t-shift as shown in S8E Fig).

These results suggested, that Exd has a role in Hox mediated pNBs apoptosis in both abdominal and Dfd-SEG regions, and perhaps this role is independent of Hth.

### Grh, AbdA and Exd bind on 1kb *F3B3*

**Fig 8:**
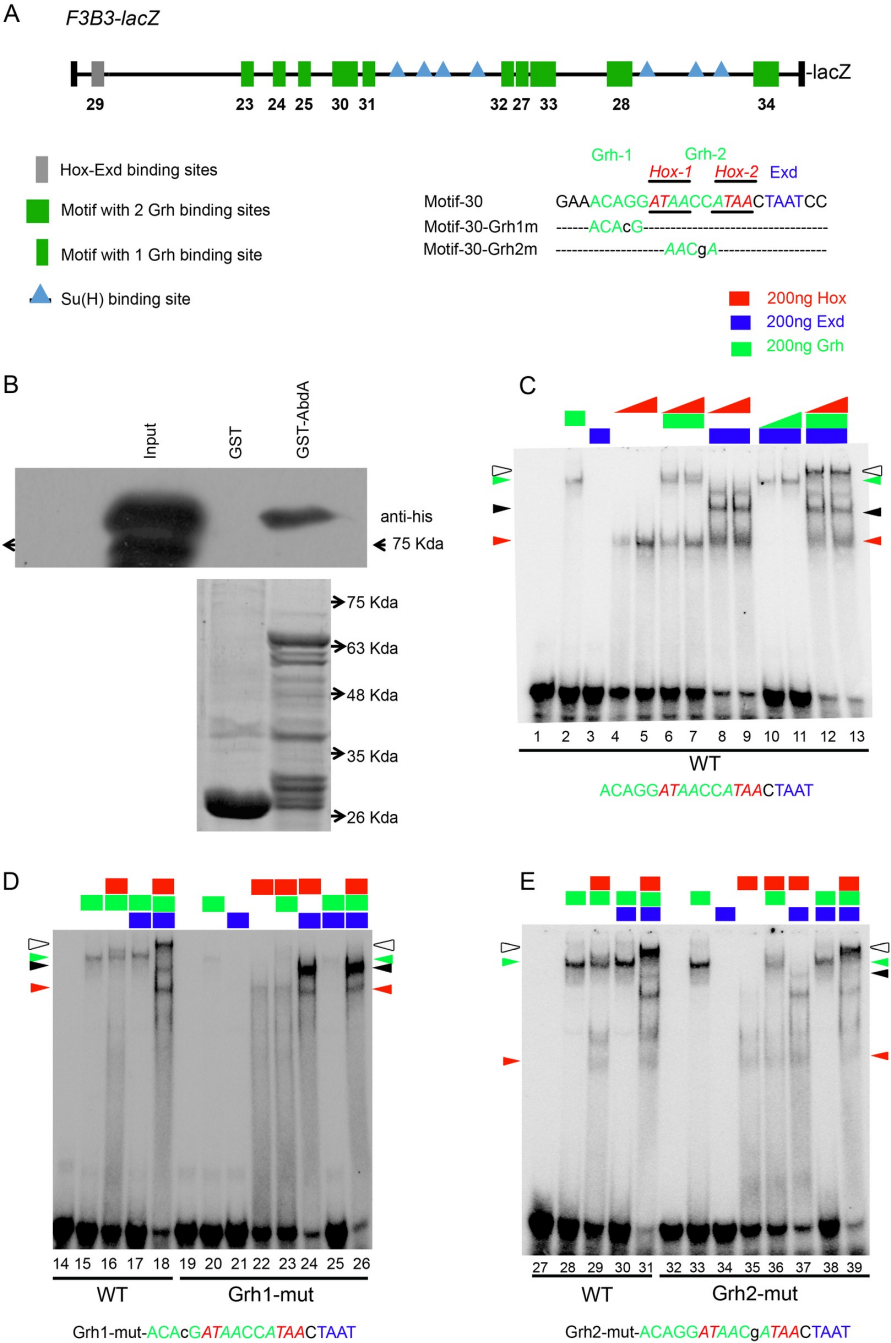
*In vitro* tetracomplex assembly on motif-30 requires Grh. (A) Schematic of 1Kb *F3B3* region. Position of Hox-Exd consensus site (A/TGATNNATNN) is shown in grey rectangle, motifs with Grh binding sites and surrounding AT rich sequences are shown as green rectangles if they have single Grh binding site and as green squares if they have two Grh binding sites. Su(H) binding sites are indicated as blue triangles. DNA Sequence for motif-30 is shown in capital letters and various mutation in this sequence are shown in small case. Hox, Exd and Grh binding sites in the sequence are shown in red, blue and green colored fonts respectively. (B) Shows western blot for GST pulldown assay showing that AbdA-Grh physically interact with each other. Anti-His antibody is used to detect His tagged Grh protein (running approximately 90Kda) pulled down by GST-AbdA. Input is His-Grh lysate. Control commassie stained gel for GST alone and GST-AbdA lanes showing comparative protein loaded in two lanes is shown below the western blot. (C) EMSA for motif-30 (lane 1–13) show that Grh and Hox bind on DNA alone (lane 2, 4–5 respectively). Hox-Grh together show a band of mobility lower than Grh alone (lane 6–7). Hox-Exd show binding (lane 8–9, black arrowhead) on DNA. Hox-Exd-Grh-DNA tetracomplex shows the lowest mobility (lane 12–13, white arrowhead). (D) EMSA with oligo mutant for Grh1 binding site (lane 19–26) show that Hox-Exd binding is intact (lane 24). Hox-Grh complex is compromised (lane16 vs 23) and tetracomplex is disrupted (lane 18 vs 26). (E) EMSA with oligo mutant for Grh2 binding site (lane 32–39) shows that Hox-Exd (lane 37) and Hox-Grh (lane 29 vs 36) complex are reduced but still present. Band for tetracomplex is slightly reduced in intensity but is intact (lane 39), indicating that Grh1 (not Grh2) binding site is critical for tetra-complex assembly. Hox binding sequence is colour coded in red (italicised and underlined as well). Grh1 and 2 binding site sequences are shown in green. Exd binding site sequence is shown in blue. Oligos used are shown at the bottom of the gel. Proteins added to a specific lane are shown at the top of the lane. Rectangles indicate constant concentrations of 200ng for Hox (red), Exd (blue) and Grh (green) respectively. Increasing concentration of 100 and 200ng for a Hox and Grh in panel-B are indicated by right triangles of red (Hox) and green (Grh) colour. Red and green arrow heads indicate Hox-DNA and Grh-DNA complex respectively. Black arrowhead indicate Hox-Exd-DNA complex. White arrowhead indicates Hox-Exd-Grh-DNA tetra-complex.

Next, we checked for potential Hox, Exd and Grh binding sites in entire 1Kb *F3B3* genomic region. Since Grh plays an important role in this apoptosis and Hox protein bind to AT rich sequences occurring at a high frequency in the genome, we decided to narrow our search for Hox sites by scanning for potential Grh binding sites in the vicinity. We identified 14 such sites conforming to variation of the known Grh binding consensus sequence (WCHGGTT) [48], these sites also had AT rich sequences (potential AbdA and Exd binding sites) in 20bp flanking region [49]. These 14 Grh sites and surrounding AT rich sequences were categorized into two type of motifs. First type only had one Grh binding site (designated as type-I), and 6 such motifs were identified (shown as green rectangles in Fig 8A). The type-II motifs had 2 closely located Grh binding sites, and 4 such motifs were identified (shown as green squares in Fig 8A). We could find only one Hox-Exd consensus site (A/TGATNNATNN) in the entire *F3B3* region referred to as motif-29 (grey rectangles, Fig 8A). We tested all these motifs for binding by EMSA. We found that 5 out of 6 Type-I motifs showed binding to Grh (motifs- 23, 25, 27, 31, 32, lanes-2, 30, 43, 82, 95, S7 Fig), motif-24 didn’t show any Grh binding (lane15-16; S7B Fig). Three out of four Type-II motifs showed Grh binding these were motif-28, 33 (lane-56 and 108, S7 Fig) and motif-30 (lane-2, Fig 8B). Motif-34 did not bind Grh. All these motifs were checked for Exd and AbdA binding as well. Motif-29 which had consensus Hox-Exd binding site showed AbdA-Exd binding but no Grh binding (lane-69, S7F Fig). The details of individual protein binding to these sites are given in S2 Table. Amongst all the motifs that were tested, we found motif-27, 30 and 32 assembled a tetracomplex (DNA-AbdA-Exd-Grh). We decided to analyze motif-30 in details since it showed a good tetracomplex formation with Grh, AbdA and Exd, as well as strong binding for each of the individual proteins.

### AbdA and Grh are important for formation of a tetracomplex on DNA

To gain insights into the tetracomplex assembly, we started out by testing motif-30 for AbdA, Grh and Exd binding. We used increasing concentration of Hox (100 and 200ng, lane-4 and 5, Fig 8C) and fixed concentration of Grh and Exd (200ng, lane-2 and 3, Fig 8C) and found that Grh (lane 2, Fig 8C) and Hox (lane 4 and 5, Fig 8C) bound to DNA on their own (binding indicated by green and red arrowheads), while Exd failed to bind on the DNA (lane 3, Fig 8C). On increasing AbdA in presence of fixed concentration of Grh (200ng), a band of mobility slightly lower than Grh band alone was observed (lane 6 and 7, Fig 8C). As expected increasing concentration of AbdA in presence of fixed concentration of Exd (200ng) showed AbdA-Exd complex formation on the DNA (lane 8 and 9, black arrowhead, Fig 8C). Similarly, Grh in the presence of a fixed concentration of Exd (200ng) together showed only a slight increase in Grh binding on DNA (lane 10 and 11, Fig 8C). Importantly, when Grh and Exd concentrations were kept constant (200ng each), addition of AbdA led to a band of lowest mobility. This tetracomplex (DNA-AbdA-Exd-Grh) is shown by a white arrowhead for lane 12 and 13 (Fig 8C). All subsequent EMSA experiments were done with fixed concentration of all the proteins (200ng).

Next, in order to test the specificity of Grh binding, oligos mutant for potential Grh1 and Grh2 binding site were analyzed. We observed dramatic decrease in Grh binding in oligo with mutation for Grh1 binding site when compared to wild type oligo (lane 15 vs 20, Fig 8D). It was also noticed that AbdA-Grh complex formation on DNA was compromised (lane 16 vs 23, Fig 8D). We found that AbdA-Exd complex was unaffected (lane 24, Fig 8D) while tetracomplex could not be visualized in case of Grh1 binding site mutant oligo (lane 18 vs 26, white arrowhead, Fig 8D). This indicates that Grh1 site is critical for tetra-complex formation and plays an important role in assembly of Hox-Grh complex.

In Grh2 binding site mutant oligo, we could not find a significant decrease in Grh binding (lane 28 vs 33, green arrow head, Fig 8E). We also found that AbdA-Grh complex is slightly reduced but is still present (lane 29 vs 36). This could be attributed to two reasons, one possibility is that Grh2 binding site plays a role in AbdA-Grh complex formation; this seems unlikely since mutation of Grh1 binding site abolished the AbdA-Grh binding completely. The more likely explanation could be that since Grh2 binding site overlaps with both Hox1 and Hox2 binding sites, and therefore the mutation of Grh2 site affects the assembly of AbdA-Grh complex.

We also found that AbdA-Exd binding on oligo mutant for Grh2 binding site was reduced but was still present (lane 37, black arrowhead, Fig 8E). This could be attributed to the fact that Grh2 site also overlaps with Hox2 site and hence could affect AbdA-Exd binding which happens on Hox2-Exd site (confirmed in later analysis in next section). Most importantly we found that tetracomplex was still intact on the oligo mutant for Grh2 binding site (lane 31 and 39, white arrowhead, Fig 8E) suggesting that Grh1 site is more important for the assembly of tetracomplex. These results suggested to us that AbdA and Grh might interact with each other physically.

To test this idea, we performed an *in vitro* GST-pull down assay, wherein bacterially expressed GST and GST-AbdA protein were bound to GST beads and incubated with His-Grh bacterial lysate (input). The proteins pulled down (from His-Grh lysate) by GST-AbdA and GST alone were separated on SDS-PAGE and probed with anti-His antibody. We found that while GST alone showed no band, GST-AbdA could successfully pull down His-Grh (approximately 90Kda- Fig 8B).

These results indicate that AbdA and Grh are not only important for tetracomplex formation, they also physically interact with each other.

### AbdA-Exd are critical for tetracomplex formation

**Fig 9:**
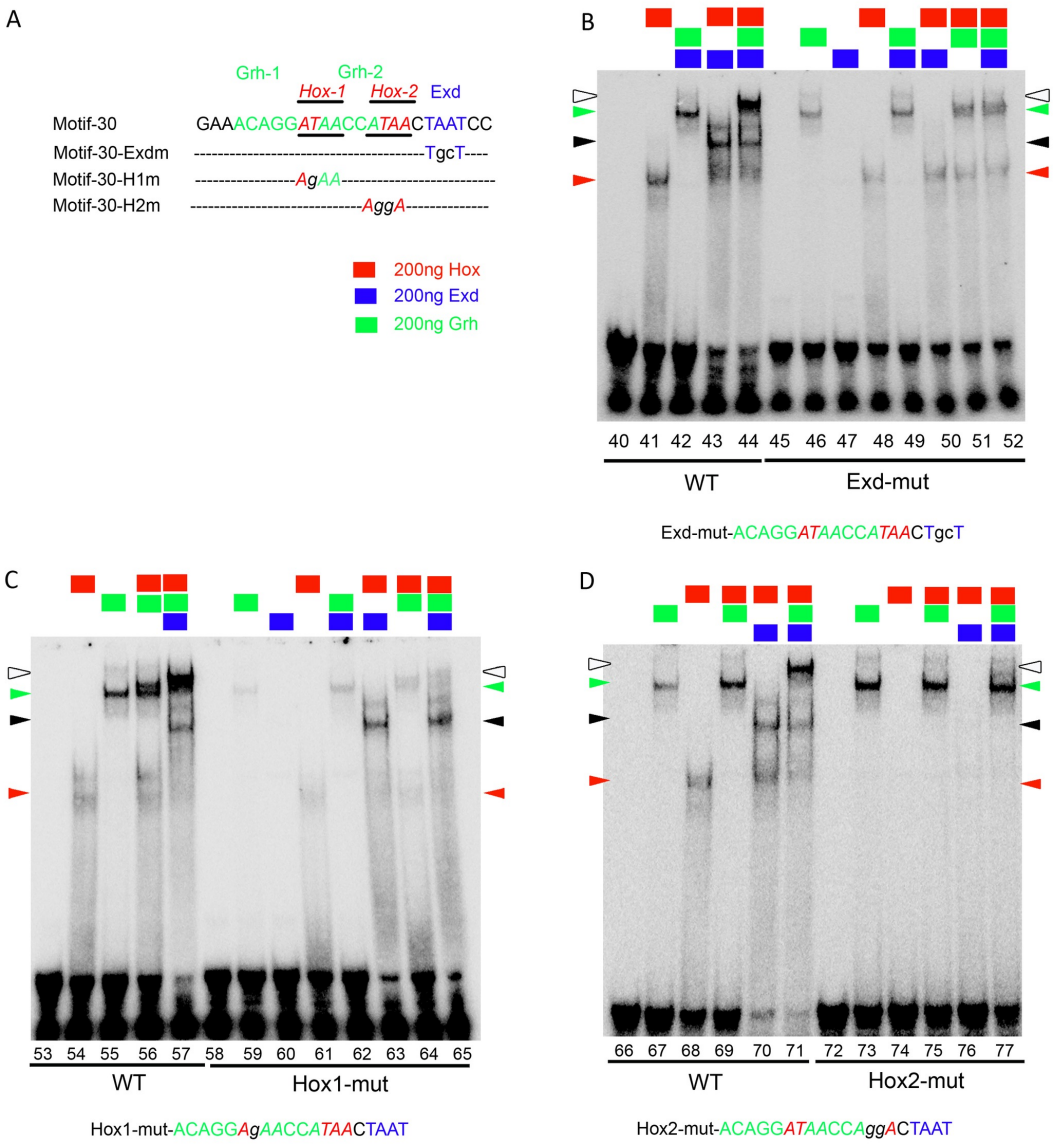
AbdA-Exd are critical for tetracomplex assembly on motif-30. (A) DNA Sequence for motif-30 is shown in capital letters and various mutation in this sequence are shown in small case. (B) EMSA with Exd binding site mutant oligo (45–52) show disruption of Hox-Exd complex (lane 50) and tetra-complex (lane 52), while Grh binding (lane 46) and Hox-Grh binding are still present (lane 51). (C) EMSA with oligo mutant for Hox1 binding site (lane 58–65) show that Hox-Exd (lane 63) and Hox-Grh binding is slightly reduced (lane 56 vs 64) but tetra-complex is dramatically reduced (lane 57 vs 65). (D) EMSA with oligo mutant for Hox2 binding sites (lane 72–77) show that Hox binding is completely disrupted on DNA (lane 74) while Grh binding on DNA is normal (lane 73) and tetracomplex binding is largely abolished (lane 77).

Next, we examined oligos mutants for AT rich sequences to identify Hox and Exd binding sites. Motif-30 oligo mutant for potential Hox1+2 binding sites showed no AbdA binding (lane 122 vs 128, S7J Fig), suggesting that these were Hox binding sites. Subsequently, we tested AbdA and Exd binding on oligo mutant for potential Exd binding site. We found that a lower mobility complex was formed by AbdA in presence of Exd protein on wild type oligo (lane 41 vs 43, Fig 9B) which was abolished in oligo mutant for Exd binding site (lane-50, Fig 9B, Exd site is shown in blue). This suggested that AbdA-Exd complex most likely assembles on Hox2-Exd sites, more so considering the proximity of the two sites. Mutation of Exd site also abrogated tetracomplex (lane 44 vs 52, white arrowhead, Fig 9B), while Grh protein still bound to DNA (lanes 46, 51–52, Fig 9B).

In case of oligo with Hox1 binding site mutation, we noticed a decrease in AbdA binding on DNA (lane 54 vs 61, red arrowhead, Fig 9C). We also found that Grh binding (lane 55 vs 59, green arrowhead, Fig 9C) was reduced but still present. This could be due to the fact that Hox1 mutation is in middle of the Grh binding sites and therefore affected Grh binding onto DNA. Moreover, we found that AbdA-Exd complex binding (lane 63, black arrowhead, Fig 9C) was intact but tetracomplex binding was dramatically diminished (lane 57 vs 65, white arrowhead). We believe this also could be due to effect of Hox1 mutation affecting nearby Grh1 binding site (as discussed above), and hence the tetracomplex formation.

Next, we tested oligo mutant for Hox2 binding site. We found that both AbdA binding (lane 68 vs 74, Fig 9D) and AbdA-Exd binding (lane 70 vs 76, Fig 9D) were abolished in this case. Though the Grh binding could still be detected (lane 73) the tetracomplex formation was completely abolished (lane 71 vs 77, Fig 9D). Since we could not observe any AbdA-Grh complex, this suggests that AbdA-Grh complex uses Hox2-Grh1 binding site. In corroboration to this, we found that in Hox1+2 double mutant oligo, AbdA-Exd, (lane 122 vs 129, S7J Fig), AbdA-Grh (lane 124 vs 130, S7J Fig) and tetracomplex binding is completely abolished (lane 125 vs 132, S7J Fig). Also the effect on complex formation was much stronger in this case compared to individual mutants for Hox1 and Hox2 sites.

The above experiments suggest that AbdA-Exd complex is critical for the tetracomplex formation. AbdA-Exd along with Grh most likely assembles a tetracomplex on Hox2-Exd and Grh1 site on DNA. We believe that this tetracomplex could contribute to regulation of RHG genes through *F3B3* enhancer.

### AbdA, Exd, Grh and Su(H) binding sites are critical for maintenance of apoptotic enhancer activity

**Fig 10:**
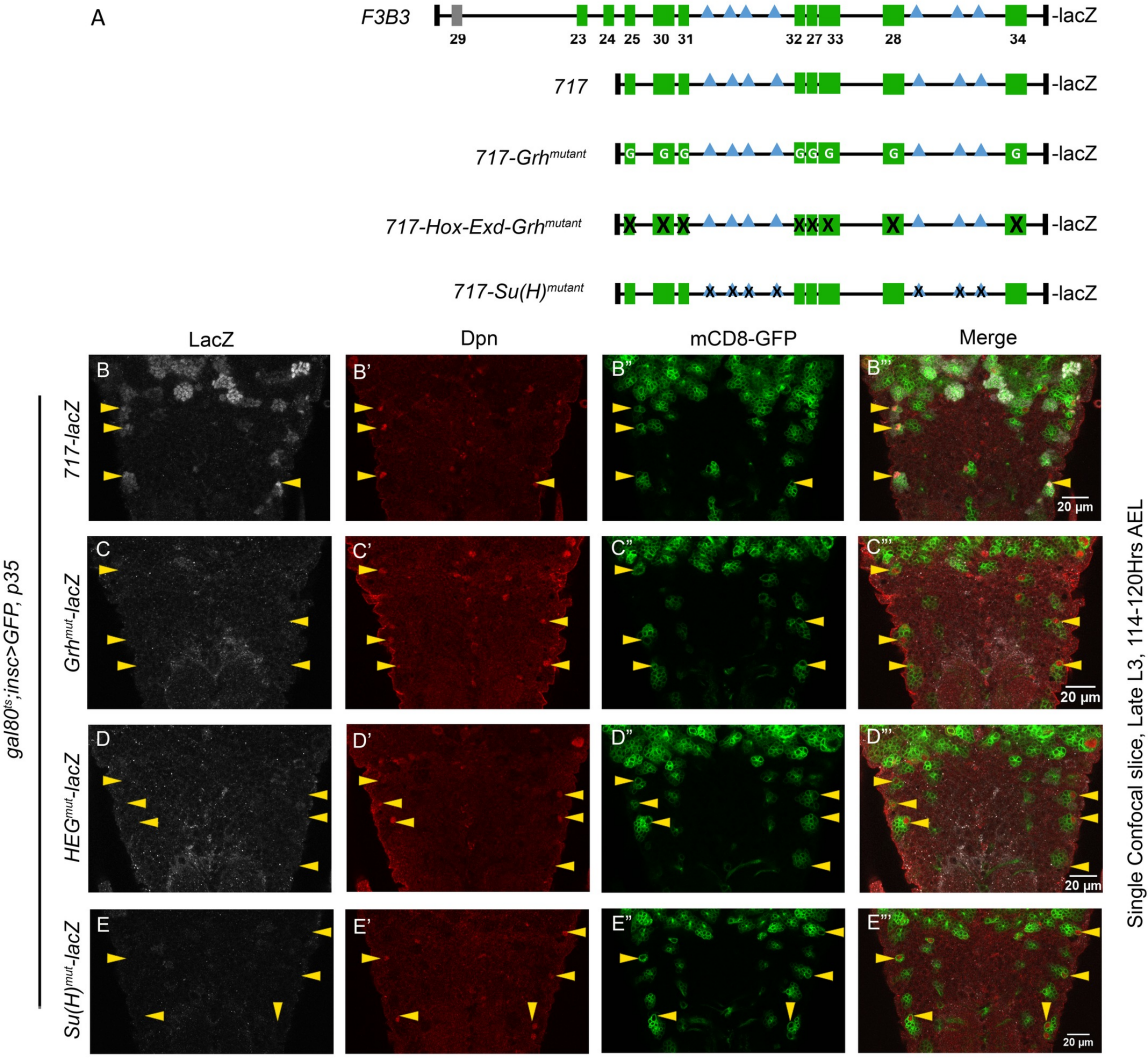
AbdA-Exd-Grh and Su(H) binding sites are required for maintenance of the enhancer. (A) Shows a comparative schematic of 1Kb *F3B3-lacZ*, *717-lacZ* and its mutant versions *717-Grh*^*mutant*^*-lacZ*, *717-HEG*^*mutant*^*-lacZ and 717-Su(H)*^*mutant*^*-lacZ*. (B) Vl pNBs resulting from blocking of death by expression of p35 in L1 stage (t-shift as shown in S8A Fig) shows expression of *717-lacZ* in late L3 stage. (C-E) Mutant versions *717-Grh*^*mutant*^*-lacZ* (C), *717-HEG*^*mutant*^*-lacZ* (D) *and 717-Su(H)*^*mutant*^*-lacZ* (E) show no lacZ expression at the same stage. Yellow arrow arrowheads indicate Vl-pNBs. “G” written within green motifs indicate that only Grh binding sites found in the motif are mutated. “X” sign on green motifs indicate that all Hox-Exd and Grh binding sites found within motif are mutated. “X” sign within blue [Su(H)] motifs indicate that Su(H) binding site is mutated. Details of specific mutation are given in S1 Text.

In order to test the *in vivo* relevance of various motif tested for AbdA, Exd and Grh binding *in vitro*, enhancer mutagenesis was carried out. All the mutagenesis studies were carried on the 717 bp subfragment (Fig 10A). Three kind of mutant constructs were made. In first construct, Grh binding sites in all 8 motifs (present in 717 bp) were mutagenized leaving Hox-Exd binding sites mostly intact (*717-Grh*^*mutant*^*-lacZ*). In the second construct Hox-Exd and Grh binding sites across all the 8 motifs were mutagenized (*717-Hox-Exd-Grh*^*mutant*^*-lacZ*) (Fig 10A). A third construct was designed to test direct role of Notch signaling in abdominal pNB apoptosis. Since Notch intracellular domain goes into the nucleus and activates gene through its executive TF Suppressor of Hairless (Su(H)), we identified and mutagenized all recognizable Su(H) binding sites in 717 bp enhancer (*717-Su(H)*^*mutant*^*-lacZ*) (Fig 10A). We could identify seven such binding sites which were variations of known consensus binding sequence for Su(H) (RTGRGAR) [50]. All the transgenic lines were crossed into the background of *UAS-p35* and were subsequently checked for the expression of reporter lacZ in abdominal pNBs in late L3 stage. For comparison of lacZ levels *tubulin-GAL80*^*ts*^*; inscGAL4* was used to drive the expression of p35 to block the apoptosis of the pNBs. This helped us to visualize the sustained expression of lacZ in later stages, which serves as a hallmark for identification of abdominal apoptotic enhancer. Since the expression of *717-lacZ* was restricted only to Vl lineage in late L3 stage, we compared wild type (Fig 10B) and mutant versions (Fig 10C–10E) of the enhancer for their capacity to drive the expression of lacZ reporter in these cells. We found that reporter lacZ expression was completely missing in abdominal Vl pNBs of all the three mutant versions of the enhancer in late L3 stage (Fig 10C–10E). Next, we decided to visualize the expression of the reporter in early L3 stage of development. Interestingly, we found that the mutant reporter lines for *717-Grh*^*mutant*^*-lacZ* and *717-Hox-Exd-Grh*^*mutant*^*-lacZ* expressed normally in abdominal pNBs (S10B–S10D Fig). These results suggested that motifs being analyzed here play a crucial role in sustenance of the expression of the apoptotic genes and are not critical for initiation of their expression in early stages.

In our analysis with *717-Su(H)*^*mutant*^*-lacZ*, we found that its expression in pNBs was slightly delayed (S10E–S10F Fig) in early stages, but in late L3 stage, like other mutant *enhancer-lacZ* lines, its expression was completely missing from Vl pNBs.

These results suggested that Notch signaling has a direct role in pNB apoptosis. The *enhancer-lacZ* analysis suggest that it may have a temporal role in apoptosis initiation but more importantly it seems to have a role in maintenance of the enhancer activity and hence RHG genes during apoptosis.

## Discussion

A large fraction of cell death in developing organism happens in CNS, which underlines its importance in CNS morphogenesis. The coupling of death in CNS with spatial developmental cues like Hox genes is a convenient strategy evolved by nature for patterning of neural tissues to coordinate developmental apoptosis with spatial regionalization of the organism. Therefore, it is of interest to understand the molecular details of this mechanism. We have investigated this in abdominal and Dfd-SEG region of larval CNS. We find that Hox mediated pNBs apoptosis happens through a battery of common players (Hox-Exd-Grh-Notch) perhaps using a similar mechanism, albeit through a different enhancer.

### Enhancer regulating the transcription of RHG genes in pNBs

**Fig 11:**
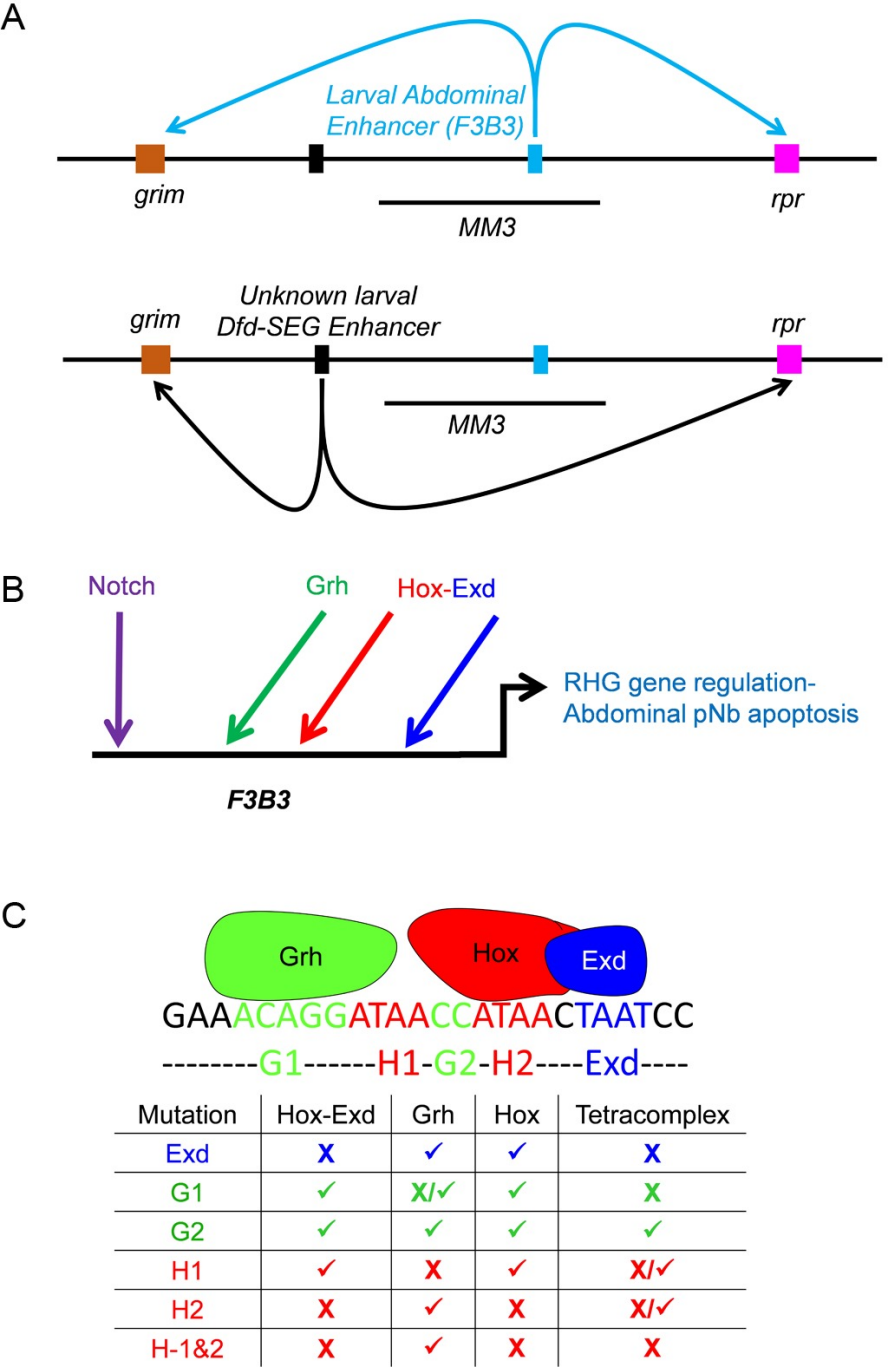
Model for pNB apoptosis. (A) Regulation of apoptotic genes *grim* and *reaper* in abdominal and Dfd-SEG happen through two distinct enhancers. Enhancer for Dfd-SEG is yet to be identified but lies outside *MM3* and is arbitrarily shown 5’ to *MM3*. (B) Model for regulation of RHG genes in abdominal pNBs. Grh, Hox-Exd and Notch signaling play a direct role in regulation of RHG genes through 1Kb *F3B3* enhancer. (C) An approximate schematic suggesting possible assembly of AbdA-Exd-Grh-DNA tetracomplex on motif-30. The Hox, Exd and Grh binding sites are indicated as H1, H2, G1, G2 and Exd. The table below the schematic is for various mutant oligos and has (X) indicating that binding is abrogated, (√) indicating the binding is present and (X/√) indicating that binding is reduced.

Previous report suggest that RHG genes (mainly *grim* and *reaper*) express and function in a combinatorial manner in dying pNBs [38]. Wherein *rpr* deletion alone shows no block of apoptosis, *grim* deletion alone shows a delay till late L3 stage, while double deletion completely block this cell death. This indicates that *grim* is the major player and *rpr* probably takes over in absence of *grim*. Since abdominal pNBs are destined to die, therefore, regulation of these genes is designed to ensure that once their expression is switched on, it should be maintained in these cells till they undergo apoptosis. It is also expected that their coordinated expression in a cells of a specific region may be regulated by a single shared enhancer [25,38]. Similarly, their expression in different regions of developing CNS may be controlled by multiple region-specific enhancers. Our data support these ideas in a limited context of *Drosophila* NBs (Fig 11A). We find that the larval abdominal pNB apoptosis is regulated by an enhancer lying within 1kb region (*F3B3*) of *NBRR*. This 1 Kb region is a subfragment of 5 Kb embryonic NB specific apoptotic enhancer (also known as *enh-1* [25]) and is deleted in *M22* (14.5 kb deletion). Our failure to recover ectopic pNBs in Dfd-SEG in *M22/MM3*, *MM3/XR38* and *MM3/MM3* combinations (Fig 5C, bars 6 & 7 of the graph) indicate that enhancer responsible for Dfd mediated pNB apoptosis (in Dfd-SEG) lies outside 22 Kb *NBRR* and 54 kb *MM3* deletion. Thus, apoptosis of larval pNBs in abdominal and Dfd-SEG region are controlled through two distinct enhancers (as shown in Fig 11A).

Therefore, while a single Hox gene like AbdA can activate pNB apoptosis by using same enhancer in embryonic and larval stages of development (as it happens in abdominal segments for *enh-1* [25] its subfragment *F3B3*), different regions of the developing CNS (abdominal and Dfd-SEG regions) employ different enhancers to activate RHG genes.

### AbdA, Exd, Grh binding on enhancer is important for maintenance of the expression of RHG genes

It is known that abdominal pNBs do not die in a synchronous manner. They start dying from early L3 stage and over a period of next 48 hrs different pNBs activate AbdA at different times and undergo apoptosis. We observed the same from our analysis of different *enhancer-lacZ* lines, wherein some cells show lacZ expression just prior to early L3 stage, while others express lacZ later on. We also observed that intensity of lacZ reporter in pNBs becomes stronger from early to late stages. This suggests that lacZ reporter expression in pNB can be categorized into two phases, initiation phase followed by maintenance phase of expression. In agreement to this, we find that different *enhancer-lacZ* lines (*F3*, *F4*, *F3B*, *F3B3*, and *717-lacZ*) show a sustained lacZ expression in the pNBs till late larval stages in a cell death blocked background (Fig 2, Fig 10B).

We identified 8 motifs with composite AbdA-Exd-Grh binding sites within 717bp enhancer. *In vitro* binding assay suggested that Hox-Exd and Grh form a tetracomplex on 3 out of 8 motifs analyzed by us (motif-27, 30 and 32). Of these 3 motifs we used motif-30 as a model to understand the complex assembly and found that all the three proteins (Hox, Exd and Grh) are critical for tetracomplex formation (Figs 8 and 9). In order to test the *in vivo* relevance of the composite Grh-AbdA-Exd binding sites found within different motifs of apoptotic enhancer, we mutagenized these binding sites. We tested the capacity of the resulting mutagenized enhancer to drive lacZ in abdominal pNBs. We found that, in both *717-Grh*^*mutant*^*-lacZ* and *717-HEG*^*mutant*^*-lacZ*, enhancers were normal for the initiation of the lacZ expression in pNBs (S10B–S10D Fig), but interestingly the mutant enhancers were incapable of sustaining the expression of lacZ reporter in these cells till later stages (Fig 10). This implies that these motifs play an important role in maintenance rather than initiation of RHG gene expression. In our experiment we mutagenized AbdA, Exd and Grh binding sites in all 8 motifs found in 717bp enhancer (*717-HEG*^*mutant*^*-lacZ*; Fig 10D). Since our analysis cannot discriminate whether 3 tetracomplex forming motifs are more critical compared to rest of the 5, therefore the results does not imply that tetracomplex is central for the maintenance activity of the apoptotic enhancer. But considering the direct physical interaction of Grh with AbdA (Fig 8B) and binding assays wherein majority of sites show Abd-Exd and AbdA-Grh complex formation (Figs 8 and 9 and S7 Fig), we believe that AbdA, Exd and Grh proteins together play a role in maintenance activity of the enhancer and a part of the same may be contributed by tetracomplexes assembled on the enhancer. Interaction of helix-loop-helix (HLH) protein (Grh in this study) and HD containing TFs (AbdA in this study) have been reported earlier. It has been shown that HLH and HD transcription factor (Meis/Prep and Pitx family) interact with each other to synergize the transcriptional response [51,52]. However, Hox per se had not been shown to interact with HLH proteins so far.

In our analysis, we have focused on Grh binding sites with nearby AT rich sequences. Some of these sequences turned out to be Hox-Exd binding sites (Fig 8C–8E, motif-30 and S7 Fig), which did not fit conventional consensus sequence A/TGATNNATNN. Therefore, it will be of interest to find out which of the domains known to be important for AbdA-Exd interaction (like YPWM and UbdA motifs [53,54]) play an important role for the complex formation on motif-30. Also, whether any of these domains will contribute to AbdA’s interaction with Grh as well [53,55]. Since our results suggest that above mentioned motifs are important for maintenance but not initiation of the enhancer activity, DNA motifs necessary for enhancer initiation are yet to be identified. We observed multiple standalone Hox binding sites in 717bp enhancer with no recognizable Grh and Exd binding sites in vicinity. These individual Hox binding sites were intact in all the three mutant versions of the enhancer tested by lacZ reporter assay. We believe that these standalone Hox sites could be the first ones to be occupied in response to increasing levels of AbdA in pNBs, and help in initiating the expression of RHG genes. Subsequently, AbdA-Exd could get recruited on the composite sites (Grh-AbdA-Exd sites) and helps to maintain the levels of RHG genes which eventually lead to death of pNBs. Since Grh is proposed to be responsible for installation of apoptotic competence [35], it could also be possible that it occupies its binding sites prior to AbdA pulse coming on. The idea of standalone Hox binding sites being the first responders to increasing Hox protein expression fits well with the fact that less regulation may be required at the at initial stage of enhancer firing. Therefore it is possible that at this stage individual Hox sites on the enhancer may get bound by any Hox protein. This is supported by the fact that overexpression of Abdominal-A or Antennapedia (Antp) or Ultrabithorax (Ubx) in thoracic pNBs resulted in their apoptosis [16]. The occupation of composite site of Grh-Hox-Exd come next and are important for maintenance of gene expression and eventual cell death.

### Hox-Grh code and pNB apoptosis in abdominal and Dfd-SEG

So far, role of Grh has been reported in cell proliferation and in installing the competence to undergo AbdA mediated apoptosis. In this study we show that Grh along with AbdA contributes to transcriptional regulation of RHG genes in causing larval pNB apoptosis. This is based on the observation that Grh and AbdA knockdown downregulated apoptotic *enhancer-lacZ* (1Kb *F3B3-lacZ* reporter line, Fig 3E). Moreover, the enhancer mutagenized for Grh binding sites could not maintain its expression in late L3 stages in cell-death blocked background. This observation further supports a transcriptional role for Grh along with AbdA in RHG regulation in pNBs.

We also observe that pNBs in abdominal and Dfd-SEG express Grh but have a very low or no Hox expression (Grh^+^/Hox^-^), while their progeny show opposite expression code (Grh^-^/Hox^+^). It is observed in abdominal region (during L3 stages), that changing expression code of pNB from **Hox**^**-**^/Grh^+^ to **Hox**^**+**^/Grh^+^ (AbdA^+^/Grh^+^) results in its apoptosis. We believe that a similar theme for pNB apoptosis is being to be followed in Dfd-SEG as well, where pNBs are known to undergo Hox dependent apoptosis [24]. We find that this apoptosis is also dependent on Grh and like in abdominal segments expression of Dfd in L2 stage may change **Dfd**^**-**^/Grh^+^ state of pNBs to **Dfd**^**+**^/Grh^+^ state and cause their apoptosis. It is interesting to note that common TF code of pNBs within different region of VNCs (abdominal and Dfd-SEG) may help them to respond to similar signals (like Hox expression in pNBs) resulting in common outcome (apoptosis in this case).

We tried testing sufficiency of Hox and Grh in causing the apoptosis in different regions, by expressing Grh in Hox positive neurons. We did not see any increase in apoptosis in abdominal neurons or neuron in any other region (Dfd-SEG and thorax) of CNS. Similarly, in Dfd-SEG region overexpression of Dfd even from early embryonic stages could not cause the death of remaining 6 pNBs. This is different from the abdominal pNB which die precociously on AbdA expression or thoracic pNBs which die on expression of AbdA or Ubx or Antp [16]. This indicated that there are other molecular players in addition to Grh which are important for Hox mediated pNB apoptosis. These factors may not be same across different regions (abdominal and Dfd-SEG), but they are likely to function with Grh and contribute to apoptosis. Moreover within a set of pNBs, these factors may be expressed differentially, i.e. they may be expressed in 4 dying pNBs but not in rest of 6 pNBs within Dfd-SEG region. Hence identifying potential partners of Grh may be useful to understand how heterogeneity is generated within a population of pNBs.

### Role of Notch signaling in pNB apoptosis

Notch mutant clones generated in A1 and A2 segments of VNC very consistently downregulated AbdA (S10A Fig). This supported the earlier claim that Notch signaling could regulate the expression of AbdA in pNBs [25], but interestingly, no AbdA mediated pNB apoptosis is reported in these two segments [16,25,35]. AbdA mediated pNB apoptosis is a hallmark of A3-A7 segments [16,35], where even though we recovered ectopic pNBs (both by Notch MARCM and by RNA interference) we could not observe significant and consistent downregulation of AbdA (Fig 6E). The levels of Grh were unaffected in these pNBs (Fig 6A–6C). Simultaneous Notch knockdown and AbdA overexpression from early L1 stage (t-shift as shown in S8A Fig) blocked abdominal pNB cell death. This suggests that Notch signaling is epistatic to AbdA apoptosis happening in abdominal segments. On the other hand in thoracic segments, Notch knockdown failed to rescue AbdA induced death of some of the thoracic pNBs. Thus, we think that Notch signaling has a direct role in abdominal pNB apoptosis which seems specific for abdominal segments.

In case of *717-Su(H)*^*mutant*^*-lacZ*, we found that the initiation of the reporter lacZ was slightly delayed but more importantly the maintenance of the expression in late L3 stage was completely crippled. This led us to suggest that perhaps Notch signaling does have a direct role in apoptosis unlike what has been reported earlier [25]. Whether Notch plays a role only in maintenance or both in initiation and maintenance of the enhancer is currently not clear.

It is to be noted that all recognizable Su(H) binding sites found in 717 bp enhancer are not close (in range of 20bp) to Grh-AbdA-Exd motifs. Therefore, how does Notch signaling play a role regulation of apoptotic enhancer remains to be investigated. One possibility is that Notch signaling is involved in initiation of the expression in collaboration with individual Hox binding sites, but this still doesn’t explain its role in enhancer maintenance.

Notch knockdown results in blocking of cell death of pNBs in Dfd-SEG, similar to abdominal pNBs (Fig 6A and 6C). Considering this we think that Notch perhaps plays similar roles in apoptosis in both these regions. Since the enhancer required for the activation RHG genes in Dfd-SEG is different from abdominal enhancer, and is yet to be identified, therefore it is difficult to currently test this idea at the moment.

### Exd play a Hth independent role in pNB apoptosis

We also tested role of known Hox cofactors Exd and Hth. Interestingly, we found that while Exd plays an important role in AbdA mediated apoptosis, Hth was not critical for this function (Fig 7A and 7B). Similarly in Dfd-SEG region knockdown of Exd but not Hth resulted in ectopic pNBs (Fig 7C).

Therefore, we are inclined to believe that while Exd is important for Hox mediated pNB apoptosis in both abdominal and Dfd-SEG region, Hth is not required in this process. Since Hth function as a nuclear transporter of Exd [47], our results suggest a Hth independent mechanism to transport Exd into the nucleus in pNBs. We were unable to test this idea directly, since we could not detect Exd protein in abdominal pNBs with the available antibodies and hence localization of Exd in *hth* mutant could not be assessed.

### Notch-Hox-Exd-Grh mediated pNB apoptosis is a common mechanism

Hox mediated apoptosis of pNB is a mechanism which is used in multiple regions of developing CNS in flies [16,23,24]. We propose that Notch-Hox-Exd-Grh are a part of common machinery employed in pNBs, involved in activation of RHG genes in developing CNS. Understanding how Notch signaling coordinate this apoptosis with Hox-Exd-Grh (in abdominal and other regions), and characterization of the assembly of this multi-protein complex on DNA will be of interest in future. Grh is not expressed in post embryonic neurons, therefore whether these neurons employ overlapping players and same enhancer or have an independent mechanism for apoptosis needs further investigations.

## Materials and methods

### *Drosophila* genetics and strains

The transgenic lines for 717bp enhancer and its mutagenized forms were generated by site specific insertion [40] of the constructs at attP40-25C6.

All other transgenic reporter lines were generated by classical P-element based transgenesis. Multiple reporter lines (at least 3 independent insertions for each fragment) were tested for their expression, representative images for all the lines are shown in the figures.

The deletion line *M22* was generated by mobilization of the MiMIC element [56,57] inserted 9 Kb from 5’ region of *NBRR* (BDSC-30966) (S4 Fig). Multiple RNA interference lines from BDSC NIG and VDRC [58] were used to confirm the results wherever possible, the quantitative data presented is from RNAi stocks which are underlined: *grh*^*RNAi*^ (VDRC-101428/KK; BDSC-28820), *abdA*^*RNAi*^ (VDRC-106155/KK), *hth*^*RNAi*^ (NIG-17117-R4 and R2, VDRC-100630/KK, 12763/GD, 12764/GD, BDSC-27655), *exd*^*RNAi*^ (VDRC-7802/GD, 7803/GD, 100687/KK), *Notch*^*RNAi*^ (BDSC-27988 and 28981). *UAS-dcr2; inscGAL4 UASmCD8-GFP; tub-GAL80*^*ts*^ (J. Knoblich, [59]), *MM3* (K.White, [38]), *worniu*GAL4 (C. Doe), *grh*^*370*^, *grh*^*B37*^ (S. Bray [42]), FRT80B-*Df(3L)H99* (R. Mann), *hth*^*P2*^ (R. Mann), *exd*^*1*^ (BDSC, 3293), FRT19A-*N*^*55e11*^ (BDSC, 28813), UAS-N-intra (BDSC 52008), *UAS-p35* (DGRC, Kyoto, 108019), *UAS-AbdA* on III (R. Mann, [53]), *UAS-AbdA* on II (K. VijayRaghavan), *elav[C155]-GAL4*, UASmCD8::GFP, hsFLP1, w (BDSC, 5146), y w; *tub-GAL80*-LL9 FRT80B (BDSC, 5191), yw; FRT82B *tub-GAL80*-LL3 (BDSC, 5135), *hsflp*,FRT19A,*tubGAL80*; *tub-GAL4*,UASmCD8GFP/cyo-GFP (H. Reichert).

### Fly husbandry

Egg collection were done for 6 hrs and flies were grown at 25°C and for all temperature shift based experiments (involving *tub-GAL80*^*ts*^) 12 hr egg collection was done at 18°C. The aging was calculated as number of hours after egg laying (AEL).

### RNA interference experiments

For lacZ quantitation experiments across different knockdowns, females of *UAS-dcr2; inscGAL4 UASmCD8-GFP; tub-GAL80*^*ts*^ were crossed to *UAS-abdA*^*RNAi*^, *UAS-grh*^*RNAi*^, *UAS-Notch*^*RNAi*^ and *UAS-p35* (DGRC-108019). Egg were collected for 12hrs at 18°C and shifted to 30°C after 42hrs (approximately late embryonic stage-early L1 stage) for all the three genotypes simultaneously. The larvae were reared at 30°C for approximately 90–91 hrs (by then in late L3 stage) and were dissected in wandering stage (114–120 hr AEL, t-shift as shown in S8B Fig), processed and imaged together. Lac-Z quantification for NBs was done in abdominal region by costaining with anti-β gal, anti-AbdA and anti-Dpn antibodies. The confocal slice which showed maximum lacZ staining (mostly this slice was at the centre of pNB) was selected for the surviving pNBs in all the combinations and compared. LacZ intensity was quantitated by ZEN 2012 software. The background signal intensity observed in region of the image outside larval brain was subtracted from lacZ signal observed in pNB.

For all other RNA knockdowns, females of *UAS-dcr2; inscGAL4 UASmCD8-GFP; tub-GAL80*^*ts*^ were crossed to males of respective RNAi lines. The flies were allowed to lay eggs at 18°C for 12 hours and were shifted to 30°C at specific times. The approximate time and temperature shift (t-shift) protocols are detailed in S8 Fig. Result text in each section refers to specific t-shift protocol used.

For Hth and Exd knockdown in Dfd-SEG region, embryos were shifted immediately after egg collection to 30°C after 12 hrs of egg laying (from early embryonic stages) and dissected at late L3 (t-shift as shown in S8E Fig). For Notch knockdown in Dfd-SEG, embryos were allowed to grow at 18°C for 42 hours (from late embryonic stages) and then kept at 30°C until dissection at late L3 (t-shift as shown in S8B Fig).

For AbdA over-expression experiments, males carrying the UAS-AbdA and *enhancer-lacZ* (*F3-lacZ*, *F4-lacZ*, *F3B3-lacZ* and *717-lacZ*) transgenes were crossed to females of the genotype *UAS-dcr2; inscGal4*,*UAS-mCD8-GFP; tubGAL80*^*ts*^ and were allowed to lay eggs at 18°C for 12 hours. Eggs were reared for 6 days at 18°C (till early L3 stage) and then shifted to 30°C for 7hrs for *F3-lacZ* and *F3B3-lacZ* (12hrs for *F4-lacZ*) and dissected immediately thereafter (t-shift as shown in S8F Fig).

### Clonal analysis

MARCM clones were generated as described previously [60]. Embryos were collected over a period of six hours at 25°C and were heat-shocked at 37°C for 60 minutes every 12 hours starting from 24 hrs AEL to 96 hrs AEL and the larvae were dissected at late L3.

### Immunohistochemistry and imaging

VNCs were dissected from larvae of desired stage and fixed in 4% paraformaldehyde in 1X PBS containing 0.1% TritonX-100 for 45 minutes. The following primary antibodies were used: rabbit anti-Dpn, 1/5000; rat anti-Dpn, 1/2000; mouse anti-Dpn, 1/2000 (Bioklone, Chennai); rabbit anti-Grh, 1/2000 (Bioklone, Chennai); mouse anti-Grh, 1/2000; rabbit anti-Dfd 1/500; mouse anti-AbdA, 1/2000; mouse anti- Ubx/AbdA (FP6.87,DSHB), 1/20; rabbit anti-exd and anti-Hth (GP-52) (R. Mann) 1:100; mouse anti-exd (EXD B11M, DSHB, R. White) 1/20; rabbit anti-β-gal (Cappel) 1:1000; chicken anti-β-gal (ab9361, Abcam), 1/2000; mouse anti-NICD (C17.9C6, DSHB, Artavanis-Tsakonas, S). Secondary antibodies conjugated to Alexa fluorophores from Molecular Probes were used: AlexaFluor405 (1/250); AlexaFluor488 (1:500); AlexaFluor555 (1/1000); and AlexaFluor647 (1/500). The samples were mounted in 70% glycerol and images were acquired with Zeiss LSM 510 Meta and LSM 700 confocal microscopes and processed using ImageJ and Adobe Photoshop CS2. In all images yellow arrowheads indicate pNBs and a dotted line shows approximate thoracic (T) and abdominal (A) boundary except for Fig 3 (where it is specified the figure legends). Scale bars are shown in figures. Microsoft Excel and GraphPad Prism was used for all the data analysis (unpaired student t-test was done to check significance of the data).

### EMSA

EMSAs were performed as previously described [61]. All DNA binding experiments were performed with 6X His-tagged forms of Grh (residues 551–1333), AbdA (residues 261–590), full length Exd co-purified with HM domain of Homothorax. The following 5X binding buffer was used: 20% glycerol, 1mg/ml BSA, 2.5mM EDTA, 50mM 1M tris pH 7.5, 50mM KCl, 5mM MgCl2, 2.5mM DTT, 90 μg/ml polyDIDC. All binding reactions were set up in a 20μl volume and incubated at room temperature for 30 minutes.

### GST-pull down assay

Bacterial cultures expressing truncated GST tagged AbdA (261 to 590 aa) and His tagged Grh (551–1333 aa) were induced for two hours with 0.5mM IPTG at 18°C. Bead bound GST-AbdA and GST were incubated separately with equal amount of His-Grh lysate for 12 hours at 4°C. Pulled down and bead bound proteins were separated by denaturing SDS–PAGE and then transferred on to Polyvinylidene fluoride membranes (66543, PALL Life Sciences, Bio Trace). The membrane was blocked in 5% skimmed milk in Tris-buffered saline with 0.1% tween 20 (TBST). Primary antibodies- mouse anti-GST (sc-138, Santa Cruz Biotechnology) and mouse anti-His (H1029, Sigma- Aldrich) were diluted 1 in 5000 in 5% milk in TBST and the blot was incubated overnight at 4°C. HRP conjugated secondary antibody (Peroxidase-AffiniPure Rabbit Anti-Mouse IgG + IgM (H+L) (315-035- 048—Jackson Immunological Research Laboratory USA) (1:5000) was used. Visualization was carried out by enhanced chemiluminescence detection (34087- SuperSignal West Pico Chemiluminiscent Substrate, ThermoFisher Scientific).

## Supporting information

S1 Fig Analysis of LacZ expression of the other NBRR sub-fragments in mid L3. (A-A”) *NBRRF1-lacZ* expression is observed in some of thoracic NBs but not the abdominal NBs. (B-B”) *NBRRF2A-lacZ* is not expressed in both thoracic and abdominal NBs. (C-C”) *NBRR F2B lacZ* expression is restricted to a small number of thoracic NBs and is not observed in abdominal NBs. (D-D”) *NBRR F3B lacZ* expression is seen in abdominal NBs. (E-E””) 1 Kb *F3B3-lacZ* line inserted at attP40A-25C6, show expression restricted to Vl-NBs (yellow arrowheads) but not Vm-NBs (white arrowheads). Dotted line demarcates the thoracic and abdominal regions of the VNC. For all the fragments, a partial Z project has been represented in the image. Red channel shows anti-Dpn staining in Panel A, D and panels-B and C show anti-Grh staining. NBs that show lacZ expression are marked by yellow arrowheads and white arrowheads indicate NBs that do not show lacZ expression.
(TIF)

S2 Fig Shows a screen shot of UCSC genome browser for 1 Kb F3B3 genomic region (1016bp) located at chr3L:18,353,984–18,355,002 (dm3, release 5). Chromatin accessibility for embryonic stage 5, 9, 10 are indicated by green orange and brown bars, different TF binding sites are shown as vertical bars of different color. Bottom of the schematic shows sequence conservation of the enhancer across 11 Drosophila species. First 299 bps didn’t show good sequence conservation across multiple species. The chromatin accessibility TF binding for this region also seemed sparse, therefore last 717 bp (highlighted by red dotted box) was selected for further analysis.
(TIF)

S3 Fig pNB expression of *enhancer-lacZ* in early L3 stage. (A-C) Shows the expression of *F3-lacZ*, *F3B3-lacZ* and *717-lacZ* in early L3 stages of development at 66–72 hrs AEL (late L2- early L3), suggesting that they reflect the temporal control of RHG gene expression in abdominal pNBs. Yellow arrowhead indicate pNBs.
(TIF)

S4 Fig Ectopic NBs in abdominal region of wild type and *M22/MM3* deletion combination. A comparison of the Abd-A stained regions of late L3 larval brains of wild type control (A-A”) and the *M22*/*MM3* combination (B-B”) is shown. Deletion combination show many surviving NBs at this stage compared to wild type. Partial Z project has been shown as representative image. Dotted lines enclose abdominal region. (C) Plot showing number of NBs counted in Abd-A stained region of late L3 larval brains in wild type and various mutant combinations. “n” indicates number of mid L3 larval brains counted for each genotype. Average values are shown in bars. Error bars shown are standard deviation. (D) Genomic mapping of the *M22* deletion: A schematic representation of the *NBRR* is shown. The blue arrowhead marks the position where the MiMIC element is inserted with respect to 5’ end of *NBRR*. PCR was used to map the extent of genomic deletion as shown. Amplicons B and D (highlighted by red bars) are amplified in wild type CS but not M22/Df(3L)*H99* or MM3/ Df(3L)*H99* (deletes entire NBRR along with other regions and hence serves as negative control). On the other hand, amplicons A and C (highlighted by green bars) were amplified both in CS and M22/ Df (3L) *H99* but not in MM3/Df(3L)*H99* suggesting that these amplicons flank the M22 deletion. Based on mapping results, the *M22* deletion roughly spans from 3L: 18,348,306 to 18, 362,966 (release 6).
Amplicon A: 3L: 18,347,043..18,348,306 (1264 bp).
Amplicon B: 3L: 18,347,864..18,349,377 (1514 bp).
Amplicon C: 3L: 18,362,966..18,363,997 (1032 bp).
Amplicon D: 3L: 18,361,407..18,362,459 (1053 bp)
(TIF)

S5 Fig Apoptotic enhancers are inducible by ectopic AbdA but not by NICD overexpression. Comparison of control and Abd-A over expressed larval VNCs with *F4-lacZ* is shown. (A-A” and B-B”) Shows basal expression in thoracic NBs and ectopic *F4-lacZ* (white channel) expression in additional thoracic NBs in response to ectopic expression of AbdA in thoracic segments of CNS. (C-C”” and D-D””) Shows ectopic *F4-lacZ* and *F3-lacZ* (white channel) expression in thoracic NBs in additional cells in response to ectopic expression of AbdA in thoracic segments of CNS. These panels show both thoracic and abdominal segments of the VNCs shown in Fig 3H. (E-E” and F-F”) and (G-G” and H-H”) show basal and ectopic lacZ (white channels) expression for *F3B3-lacZ* and *717-lacZ*. *F3B3-lacZ* expression was induced as observed by high intensity of lacZ expression in thoracic NBs as well as in central brain (panel F) compared to controls which show more limited and less intense expression (panel E). In *717-lacZ* induction in response to AbdA was scored by additional lacZ positive pNBs in thoracic segment and central brain region of CNS (panel H-H”) compared to control with no ectopic AbdA expression (panel G-G”). (I) Show quantitation of Dpn intensity of abdominal NB in p35 expressing NBs (32.7+/-4.4) compared to NBs with *Notch* (24.6+/-7.8), *abdA* (27.1+/-7.5) and *grh* (27.4+/-7.8) knockdown. Average Intensity and Standard Deviation are shown in brackets. (J-J” and K-K”‘) Show that *F3-lacZ* is not induced in thoracic pNBs in response to ectopic expression of NICD. (L-L””) Shows that F3-lacZ is not induced in abdominal NBs in response to overexpression of NICD. The dotted lines in panels A-D enclose abdominal segments of larval VNC which normally express Abd-A. While in rest of the panels dotted line indicates separation of thoracic and abdominal segments. Yellow arrowheads indicate pNBs. Abdominal and Thoracic segments are indicated as “A” and “T”.
(TIF)

S6 Fig Thoracic and abdominal pNBs with AbdA and Notch knockdown. (A-A”‘, B-B”‘ and C-C”‘) Shows abdominal and thoracic pNBs with Notch knockdown and overexpression of AbdA in mid L3 stage of development. Panel-B-B”‘ are inset of panel-A, and shows three ectopic pNBs in abdominal region at higher magnification. A” and B” show expression of AbdA in pNBs. (D-D”‘) Show thoracic pNBs with Notch knockdown and overexpression of AbdA in late L3 stage of development. Yellow arrowheads indicate pNBs. Abdominal and Thoracic segments are indicated as “A” and “T” and separated by a white horizontal bar (in A-A””).
(TIF)

S7 Fig Analysis of Hox, Exd and Grh binding on motifs from 1kb *F3B3*. (A) Grh binds to motif-23 (lane2) which also shows the formation of a Hox-Exd complex (lanes 10, 11) but no higher shift is observed upon the addition of Grh to this complex.(B) Grh does not bind to motif-24 (lanes 15, 16) but a strong Hox-Exd assembles on it (lanes 23, 24) even though Exd (lanes 17, 18) and Hox alone (lanes 19, 20) show no significant binding. (C) Both Grh (lane 30) and Hox-Exd (lanes 38, 39) assemble on motif-25 but no tetracomplex results upon the addition of all the three proteins (lanes 40, 41). (D) Motif-27 show both Grh binding (lane 43) and Hox-Exd complex formation (lanes 51, 52) and addition of all the three leads to the formation of a tetracomplex (lanes 53, 54.) (E) No tetracomplex formation is seen in case of motif-28 (lane 66, 67), though both Grh (lane 56) and Hox-Exd (lane 63, 64) bind to it. Addition of Hox to Grh results in the formation of a lower mobility complex (lane 60, 61). (F) Grh fails to bind to motif-29 oligo (lane 69) but Hox-Exd complex show a strong binding on the same (Lane 77, 78). Predictably, addition of Grh does not alter the complex formation (lanes 79, 80). (G) Both Grh (lane 82) and Hox (lane 84, 85) bind to motif-31 and a Hox-Exd complex is also detected (lane 90, 91) but the presence of all the three proteins is not sufficient for the tetracomplex to form (lanes 92, 93). (H) In case of motif-32, Hox alone shows negligible binding but along with Exd, a higher complex forms (lanes 103, 104). Exd alone also binds to this site (lane 96) and a tetracomplex assembles when all the three proteins are added (lanes 105,106). (I) Grh (lane 108), Exd (lane 109) and Hox (lane 110,111) are individually able to bind on motif-33 containing oligo. AbdA-Exd and AbdA-Grh complex can be seen (lanes 116, 117 and lanes 112-113, respectively) but tetracomplex does not form on this site (lanes 118, 119). (J) Mutating both Hox sites in motif-30 results in complete loss of Hox-Exd binding (lanes 128, compare with lane 122) as well as partial loss of Grh binding (Lane 127 vs 121). As a result, the tetracomplex is entirely disrupted (Lane 132, compare with lane 125). Proteins added to a specific lane are shown at the top of the lane. Rectangles indicate constant concentrations of 200ng for Hox (red), Exd (blue) and Grh (green) respectively. Increasing concentration of 100 and 200ng for a Hox and Grh in panel-B are indicated by right triangles of red (Hox) and green (Grh) colour. Red and green arrow heads indicate Hox-DNA and Grh-DNA complex respectively. Black arrowhead indicate Hox-Exd-DNA complex. White arrowhead indicates Hox-Exd-Grh-DNA tetra-complex. The sequence of oligo tested for all these motif are given below the gels, potential Grh binding site is highlighted in green and AT rich sequences are highlighted in red.
(TIF)

S8 Fig Approximate timings for different temperature shift protocol used in different experiments are shown. Downward facing arrow indicates time of dissection.
(TIF)

S9 Fig Hth protein knockdown in thoracic lineage by RNA interference. (A-A”‘) Shows normal expression of Hth protein in thoracic lineages of control VNC at late L3 stage of development. (B-B”‘) Show knockdown of Hth protein in thoracic lineages of Hth RNA interference line (NIG-17117-R4). The knockdown was initiated from early embryonic stages. Staining of Hth in some cells outside GFP marked lineages in thoracic segments suggest that Hth staining has worked.
(TIF)

S10 Fig (A-A”‘) AbdA is down regulated in *Notch*^*55e11*^ clones in A1-A2 segments. (B-D) VNCs of *717-lacZ*, *717-Grh*^*mutant*^*-lacZ*, *717-HEG*^*mutant*^*-lacZ* show normal expression in abdominal NBs in early L3 stage. (E-F) Abdominal pNBs of VNCs of *717-Su(H)*^*mutant*^*-lacZ* do not express in early L3 stage (E), but the expression comes on in mid L3 stage (F). pNBs are indicated by yellow arrowheads.
(TIF)

S1 Text Details of M22 deletion. Detailed method to identify ectopic NBs in Dfd expressing region of SEG. Sequence of wild type and mutant binding motifs in 717bp enhancer.
(DOCX)

S1 Table Summarizes mid L3 stage expression of all the enhancer fragments and subfragments in different region of larval CNS. (DOCX)

S2 Table Summarizes the binding details of all the 10 motifs analyzed for individual proteins and the combinations analyzed. (DOCX)
