# Supplementary figures and images for: Combinatorial action of Grainyhead, Extradenticle and Notch in regulating Hox mediated apoptosis in Drosophila larval CNS
Source: PLoS Genet. 2017 Oct 12;13(10):e1007043. doi: 10.1371/journal.pgen.1007043 (PMC5667929; doi:10.1371/journal.pgen.1007043)

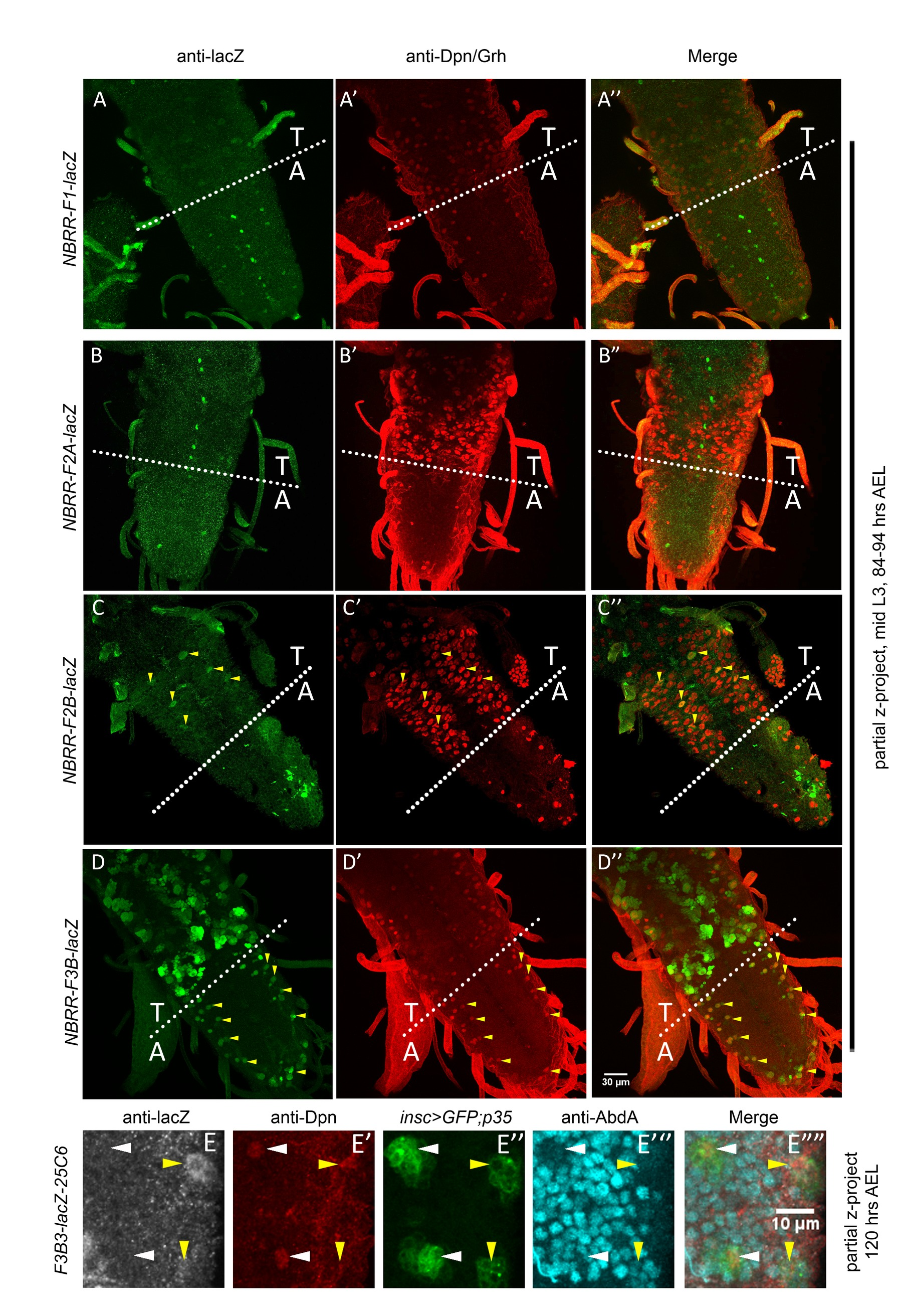

Supplement: S1 Fig — (A-A”) NBRRF1-lacZ expression is observed in some of thoracic NBs but not the abdominal NBs. (B-B”) NBRRF2A-lacZ is not expressed in both thoracic and abdominal NBs. (C-C”) NBRR F2B lacZ expression is restricted to a small number of thoracic NBs and is not observed in abdominal NBs. (D-D”) NBRR F3B lacZ expression is seen in abdominal NBs. (E-E””) 1 Kb F3B3-lacZ line inserted at attP40A-25C6, show expression restricted to Vl-NBs (yellow arrowheads) but not Vm-NBs (white arrowheads). Dotted line demarcates the thoracic and abdominal regions of the VNC. For all the fragments, a partial Z project has been represented in the image. Red channel shows anti-Dpn staining in Panel A, D and panels-B and C show anti-Grh staining. NBs that show lacZ expression are marked by yellow arrowheads and white arrowheads indicate NBs that do not show lacZ expression. (TIF) [file pgen.1007043.s001.tif]

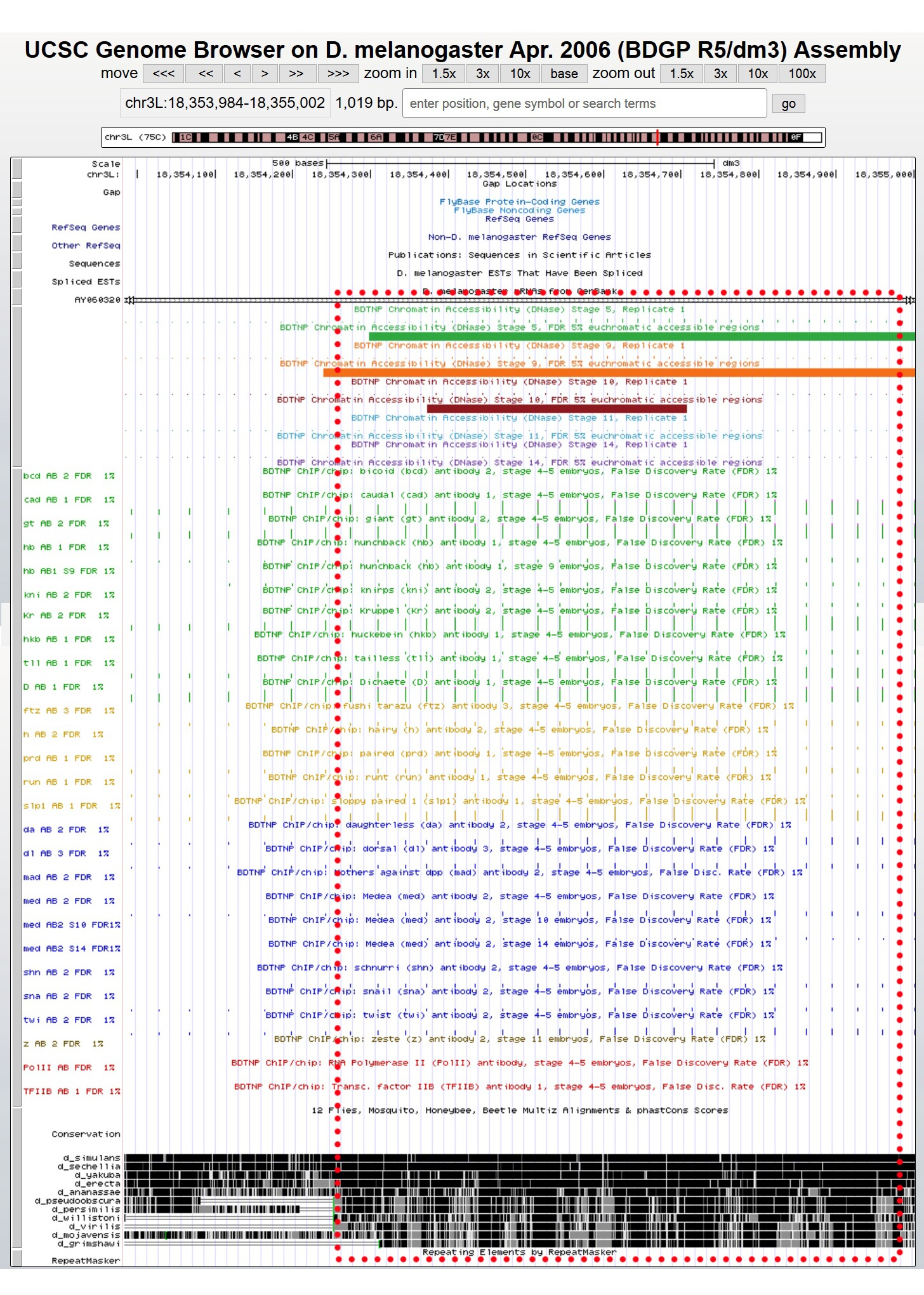

Supplement: S2 Fig — Chromatin accessibility for embryonic stage 5, 9, 10 are indicated by green orange and brown bars, different TF binding sites are shown as vertical bars of different color. Bottom of the schematic shows sequence conservation of the enhancer across 11 Drosophila species. First 299 bps didn’t show good sequence conservation across multiple species. The chromatin accessibility TF binding for this region also seemed sparse, therefore last 717 bp (highlighted by red dotted box) was selected for further analysis. (TIF) [file pgen.1007043.s002.tif]

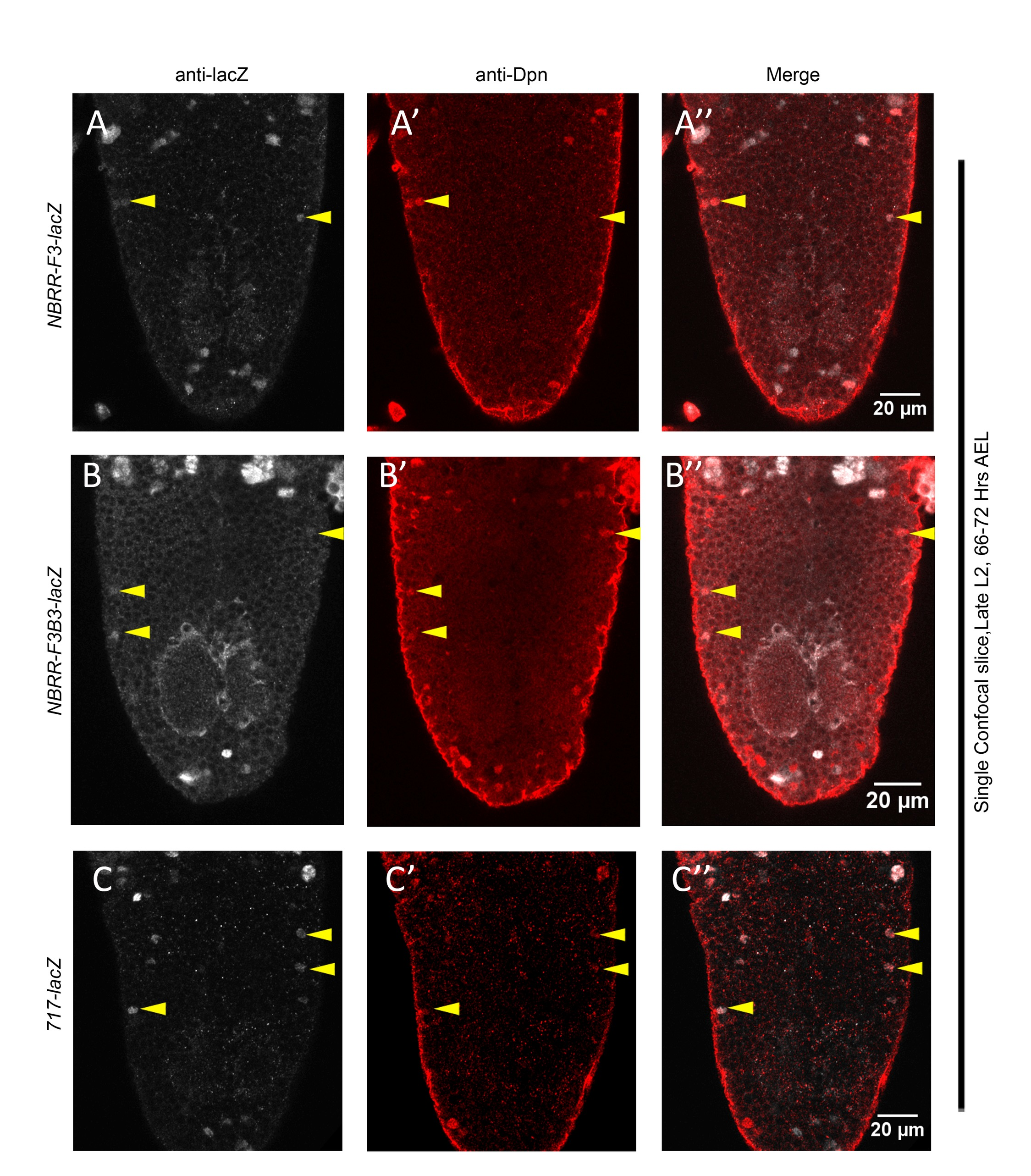

Supplement: S3 Fig — (A-C) Shows the expression of F3-lacZ, F3B3-lacZ and 717-lacZ in early L3 stages of development at 66–72 hrs AEL (late L2- early L3), suggesting that they reflect the temporal control of RHG gene expression in abdominal pNBs. Yellow arrowhead indicate pNBs. (TIF) [file pgen.1007043.s003.tif]

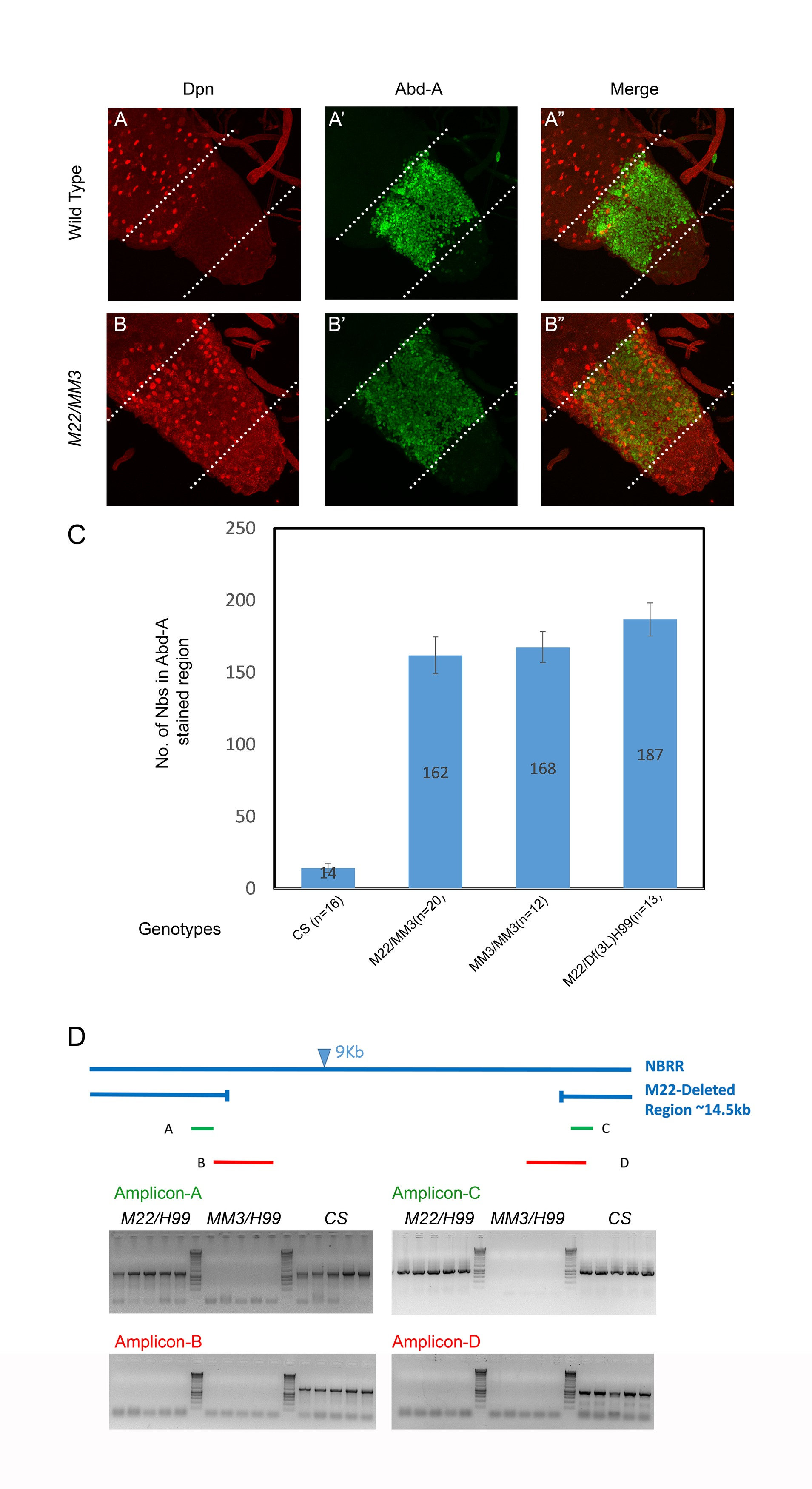

Supplement: S4 Fig — A comparison of the Abd-A stained regions of late L3 larval brains of wild type control (A-A”) and the M22/MM3 combination (B-B”) is shown. Deletion combination show many surviving NBs at this stage compared to wild type. Partial Z project has been shown as representative image. Dotted lines enclose abdominal region. (C) Plot showing number of NBs counted in Abd-A stained region of late L3 larval brains in wild type and various mutant combinations. “n” indicates number of mid L3 larval brains counted for each genotype. Average values are shown in bars. Error bars shown are standard deviation. (D) Genomic mapping of the M22 deletion: A schematic representation of the NBRR is shown. The blue arrowhead marks the position where the MiMIC element is inserted with respect to 5’ end of NBRR. PCR was used to map the extent of genomic deletion as shown. Amplicons B and D (highlighted by red bars) are amplified in wild type CS but not M22/Df(3L)H99 or MM3/ Df(3L)H99 (deletes entire NBRR along with other regions and hence serves as negative control). On the other hand, amplicons A and C (highlighted by green bars) were amplified both in CS and M22/ Df (3L) H99 but not in MM3/Df(3L)H99 suggesting that these amplicons flank the M22 deletion. Based on mapping results, the M22 deletion roughly spans from 3L: 18,348,306 to 18, 362,966 (release 6). Amplicon A: 3L: 18,347,043..18,348,306 (1264 bp). Amplicon B: 3L: 18,347,864..18,349,377 (1514 bp). Amplicon C: 3L: 18,362,966..18,363,997 (1032 bp). Amplicon D: 3L: 18,361,407..18,362,459 (1053 bp) (TIF) [file pgen.1007043.s004.tif]

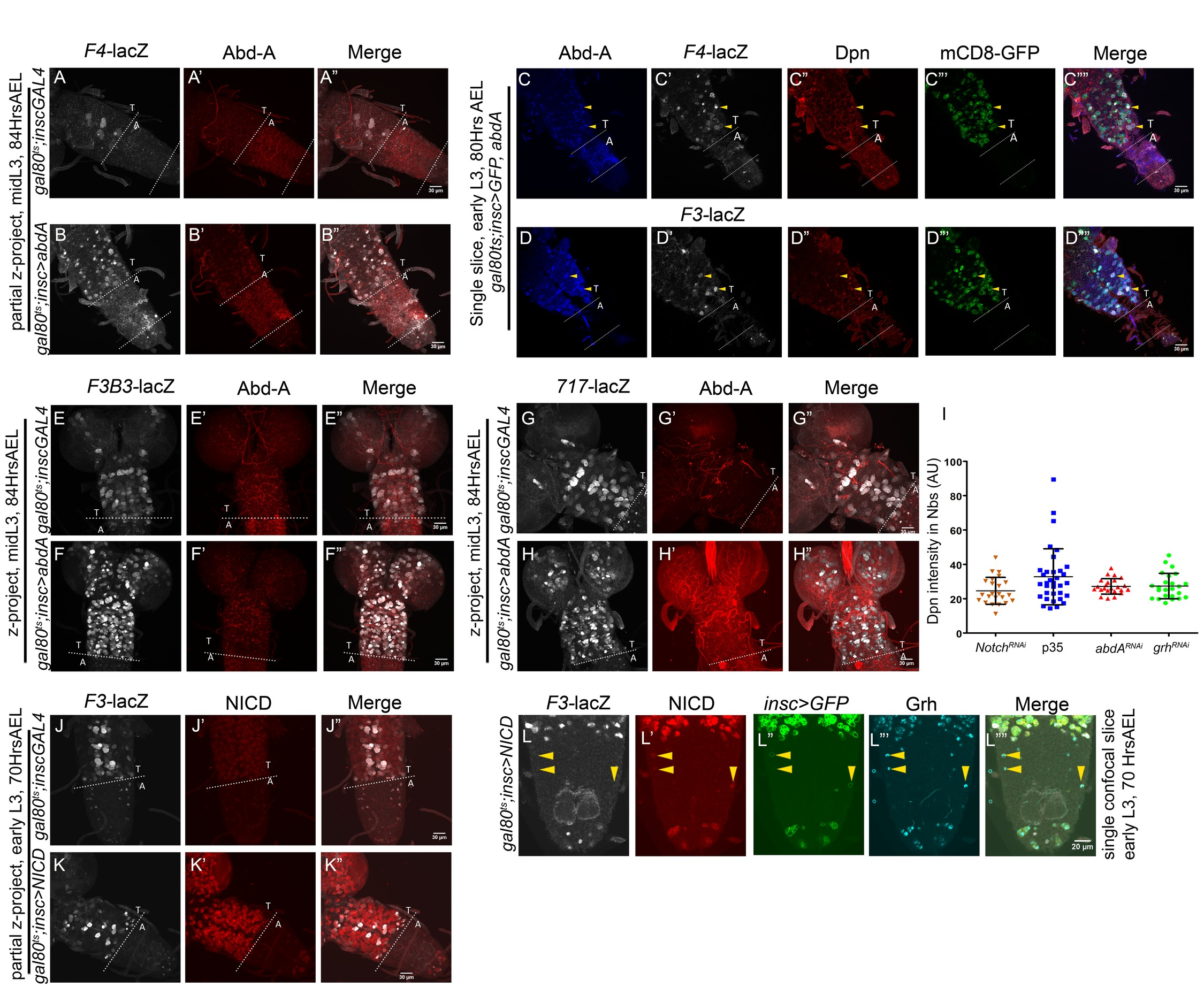

Supplement: S5 Fig — Comparison of control and Abd-A over expressed larval VNCs with F4-lacZ is shown. (A-A” and B-B”) Shows basal expression in thoracic NBs and ectopic F4-lacZ (white channel) expression in additional thoracic NBs in response to ectopic expression of AbdA in thoracic segments of CNS. (C-C”” and D-D””) Shows ectopic F4-lacZ and F3-lacZ (white channel) expression in thoracic NBs in additional cells in response to ectopic expression of AbdA in thoracic segments of CNS. These panels show both thoracic and abdominal segments of the VNCs shown in Fig 3H. (E-E” and F-F”) and (G-G” and H-H”) show basal and ectopic lacZ (white channels) expression for F3B3-lacZ and 717-lacZ. F3B3-lacZ expression was induced as observed by high intensity of lacZ expression in thoracic NBs as well as in central brain (panel F) compared to controls which show more limited and less intense expression (panel E). In 717-lacZ induction in response to AbdA was scored by additional lacZ positive pNBs in thoracic segment and central brain region of CNS (panel H-H”) compared to control with no ectopic AbdA expression (panel G-G”). (I) Show quantitation of Dpn intensity of abdominal NB in p35 expressing NBs (32.7+/-4.4) compared to NBs with Notch (24.6+/-7.8), abdA (27.1+/-7.5) and grh (27.4+/-7.8) knockdown. Average Intensity and Standard Deviation are shown in brackets. (J-J” and K-K”‘) Show that F3-lacZ is not induced in thoracic pNBs in response to ectopic expression of NICD. (L-L””) Shows that F3-lacZ is not induced in abdominal NBs in response to overexpression of NICD. The dotted lines in panels A-D enclose abdominal segments of larval VNC which normally express Abd-A. While in rest of the panels dotted line indicates separation of thoracic and abdominal segments. Yellow arrowheads indicate pNBs. Abdominal and Thoracic segments are indicated as “A” and “T”. (TIF) [file pgen.1007043.s005.tif]

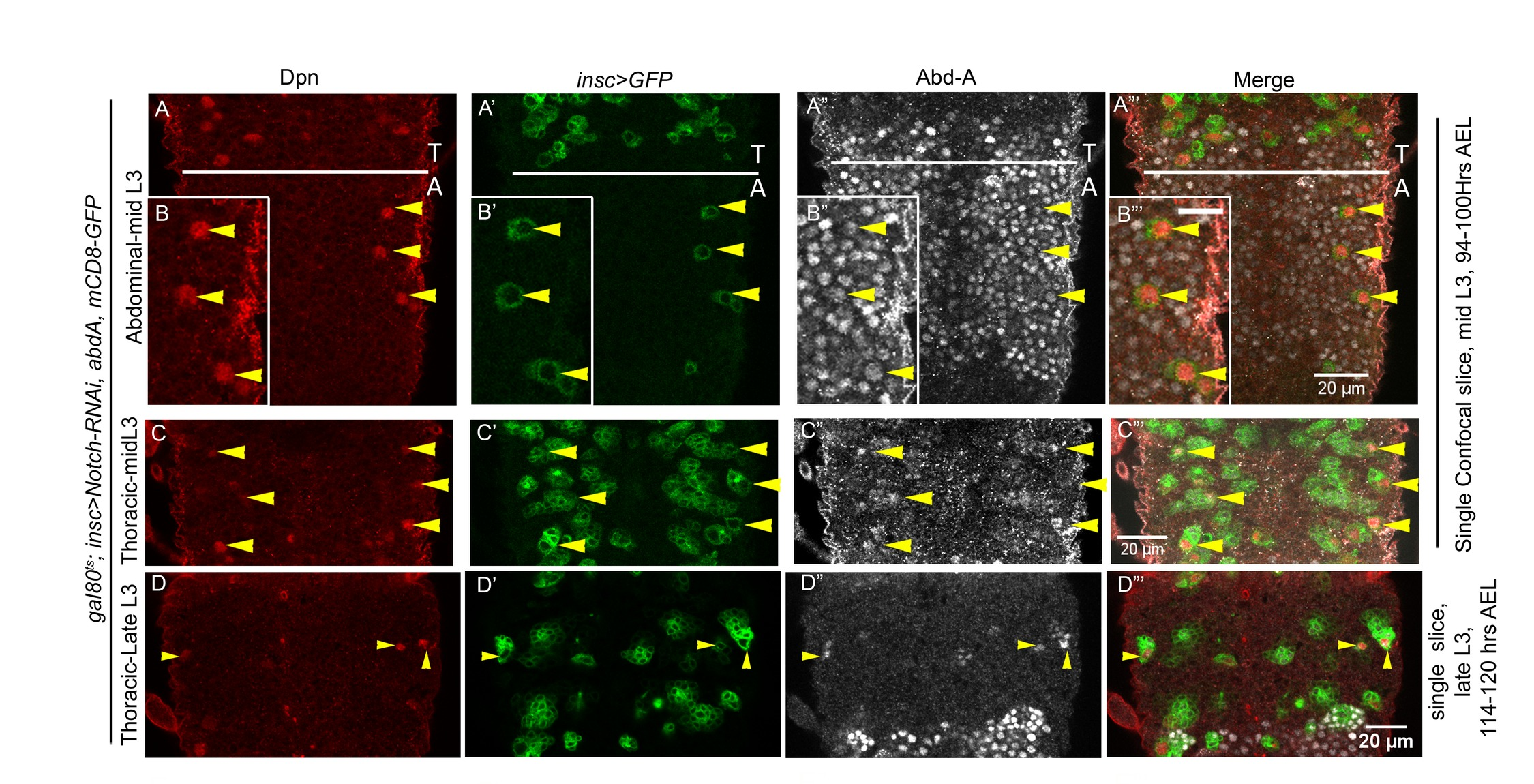

Supplement: S6 Fig — (A-A”‘, B-B”‘ and C-C”‘) Shows abdominal and thoracic pNBs with Notch knockdown and overexpression of AbdA in mid L3 stage of development. Panel-B-B”‘ are inset of panel-A, and shows three ectopic pNBs in abdominal region at higher magnification. A” and B” show expression of AbdA in pNBs. (D-D”‘) Show thoracic pNBs with Notch knockdown and overexpression of AbdA in late L3 stage of development. Yellow arrowheads indicate pNBs. Abdominal and Thoracic segments are indicated as “A” and “T” and separated by a white horizontal bar (in A-A””). (TIF) [file pgen.1007043.s006.tif]

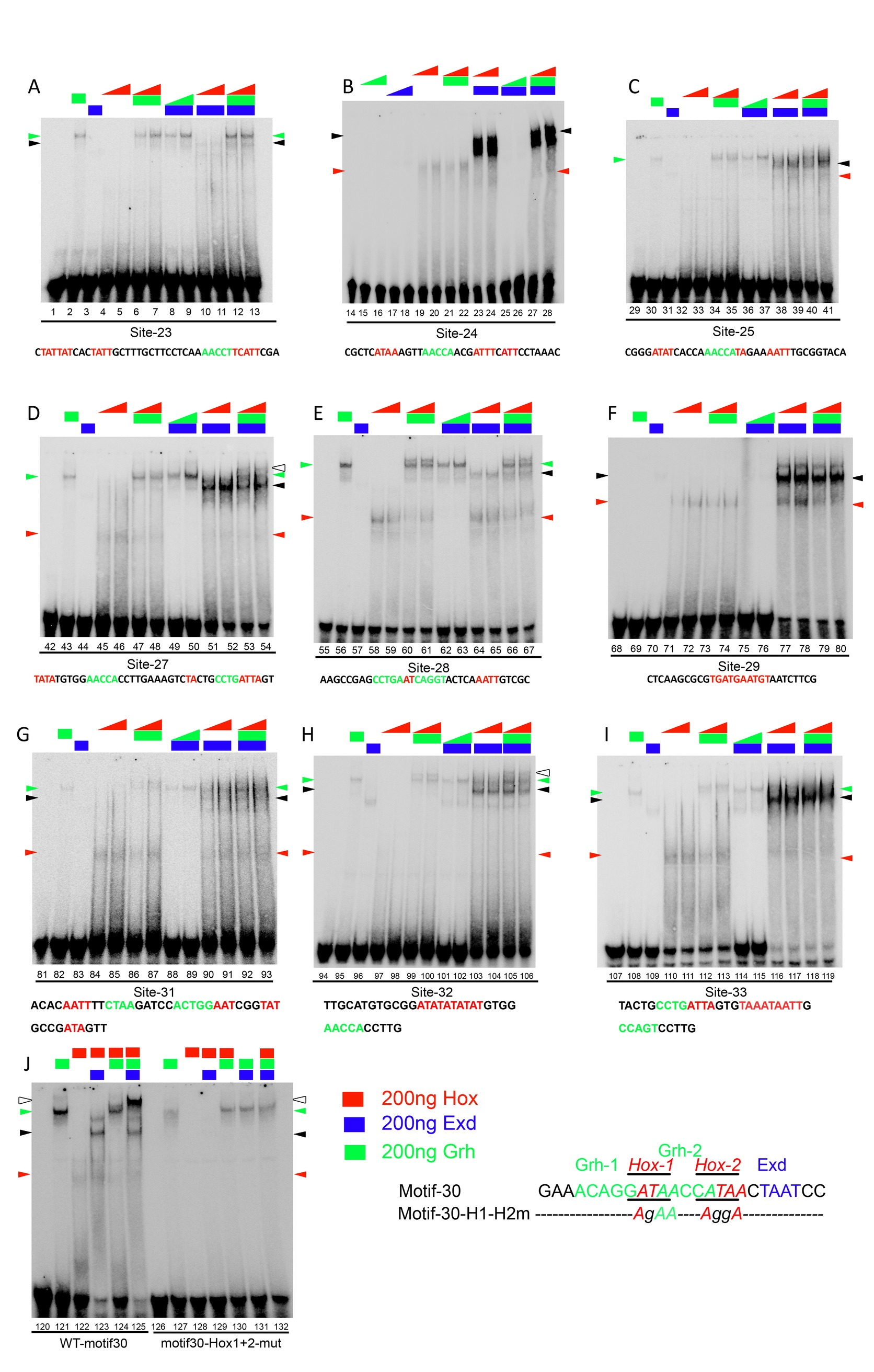

Supplement: S7 Fig — (A) Grh binds to motif-23 (lane2) which also shows the formation of a Hox-Exd complex (lanes 10, 11) but no higher shift is observed upon the addition of Grh to this complex.(B) Grh does not bind to motif-24 (lanes 15, 16) but a strong Hox-Exd assembles on it (lanes 23, 24) even though Exd (lanes 17, 18) and Hox alone (lanes 19, 20) show no significant binding. (C) Both Grh (lane 30) and Hox-Exd (lanes 38, 39) assemble on motif-25 but no tetracomplex results upon the addition of all the three proteins (lanes 40, 41). (D) Motif-27 show both Grh binding (lane 43) and Hox-Exd complex formation (lanes 51, 52) and addition of all the three leads to the formation of a tetracomplex (lanes 53, 54.) (E) No tetracomplex formation is seen in case of motif-28 (lane 66, 67), though both Grh (lane 56) and Hox-Exd (lane 63, 64) bind to it. Addition of Hox to Grh results in the formation of a lower mobility complex (lane 60, 61). (F) Grh fails to bind to motif-29 oligo (lane 69) but Hox-Exd complex show a strong binding on the same (Lane 77, 78). Predictably, addition of Grh does not alter the complex formation (lanes 79, 80). (G) Both Grh (lane 82) and Hox (lane 84, 85) bind to motif-31 and a Hox-Exd complex is also detected (lane 90, 91) but the presence of all the three proteins is not sufficient for the tetracomplex to form (lanes 92, 93). (H) In case of motif-32, Hox alone shows negligible binding but along with Exd, a higher complex forms (lanes 103, 104). Exd alone also binds to this site (lane 96) and a tetracomplex assembles when all the three proteins are added (lanes 105,106). (I) Grh (lane 108), Exd (lane 109) and Hox (lane 110,111) are individually able to bind on motif-33 containing oligo. AbdA-Exd and AbdA-Grh complex can be seen (lanes 116, 117 and lanes 112-113, respectively) but tetracomplex does not form on this site (lanes 118, 119). (J) Mutating both Hox sites in motif-30 results in complete loss of Hox-Exd binding (lanes 128, compare with lane 122) as well as [file pgen.1007043.s007.tif]

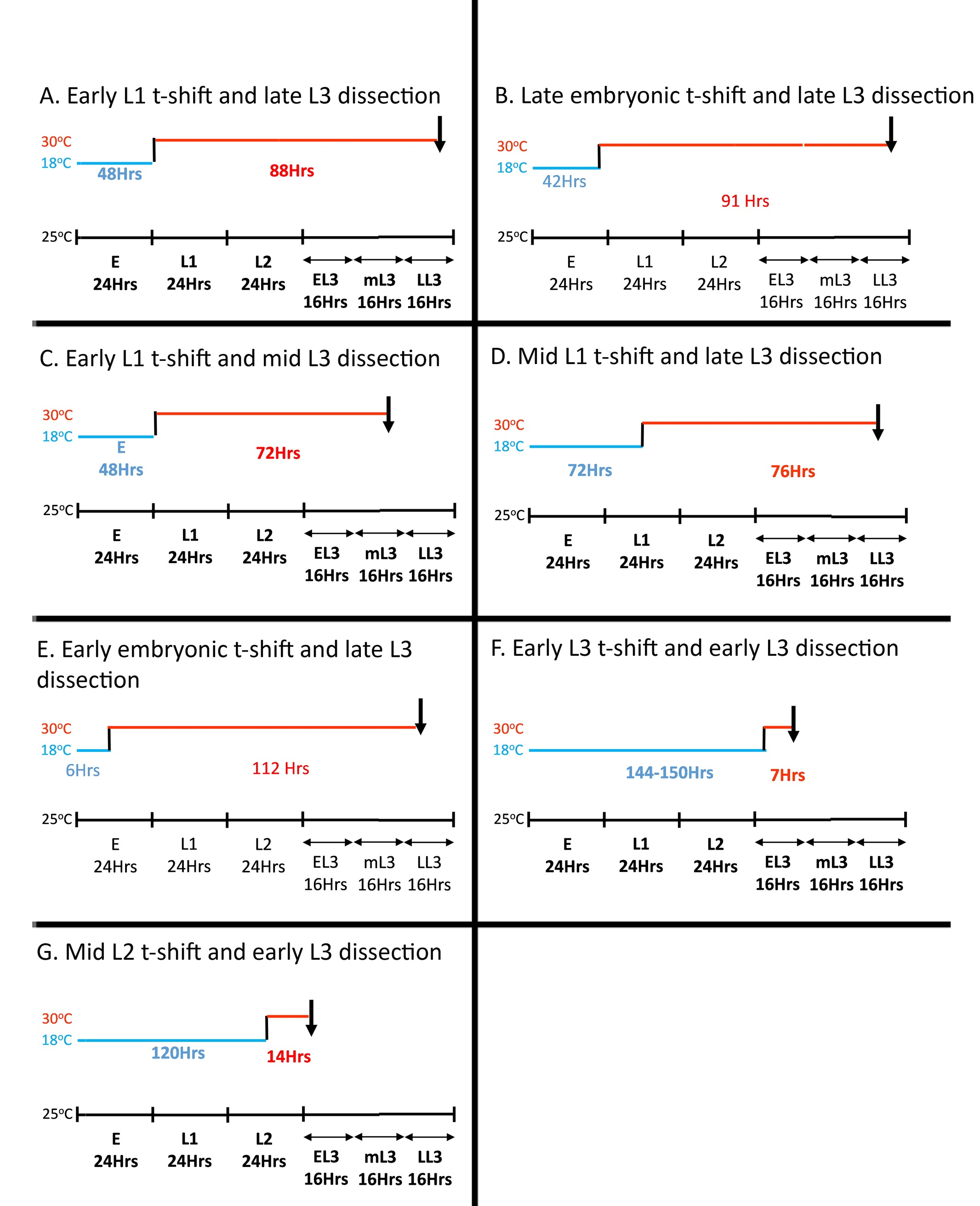

Supplement: S8 Fig — Downward facing arrow indicates time of dissection. (TIF) [file pgen.1007043.s008.tif]

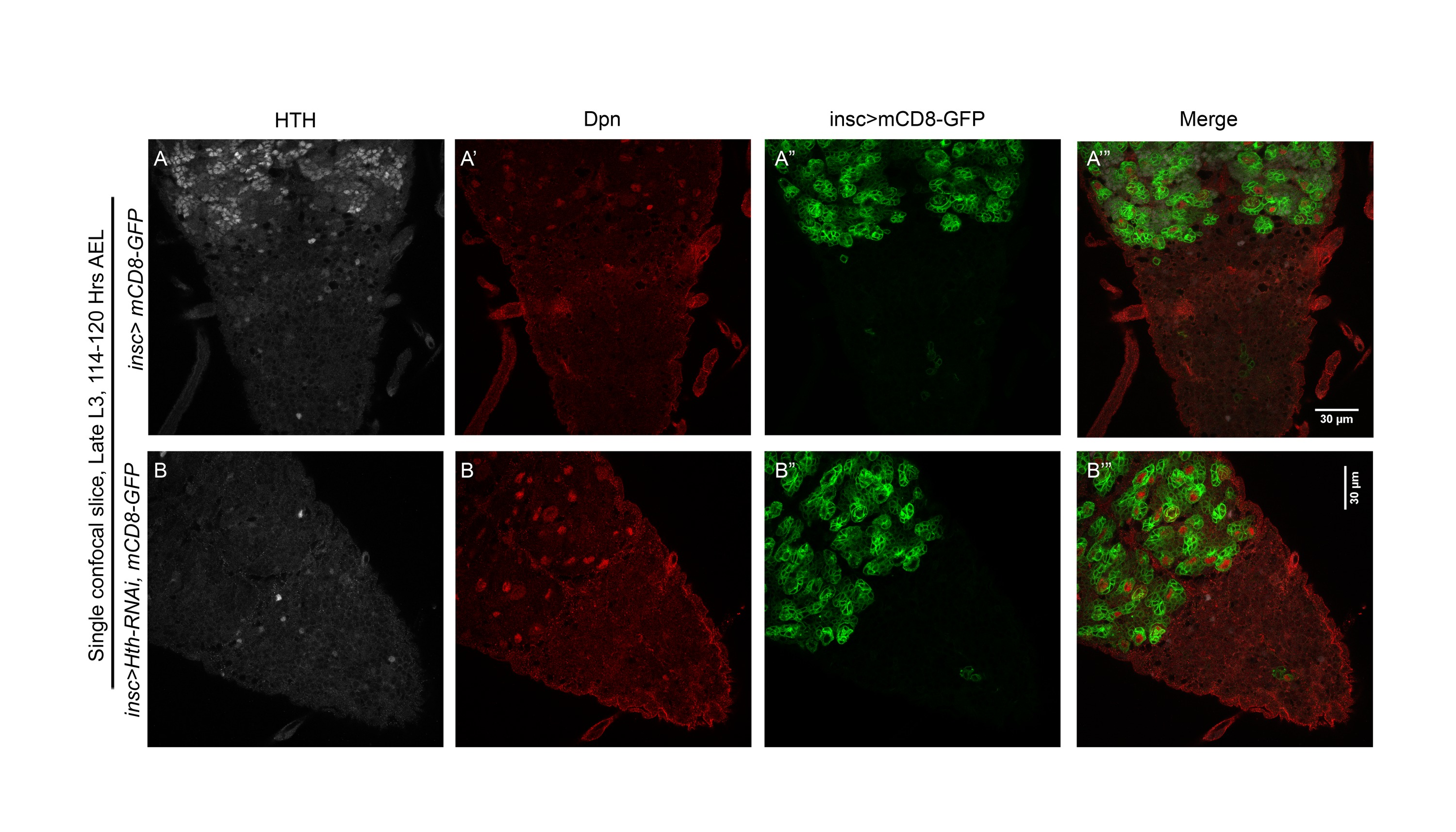

Supplement: S9 Fig — (A-A”‘) Shows normal expression of Hth protein in thoracic lineages of control VNC at late L3 stage of development. (B-B”‘) Show knockdown of Hth protein in thoracic lineages of Hth RNA interference line (NIG-17117-R4). The knockdown was initiated from early embryonic stages. Staining of Hth in some cells outside GFP marked lineages in thoracic segments suggest that Hth staining has worked. (TIF) [file pgen.1007043.s009.tif]

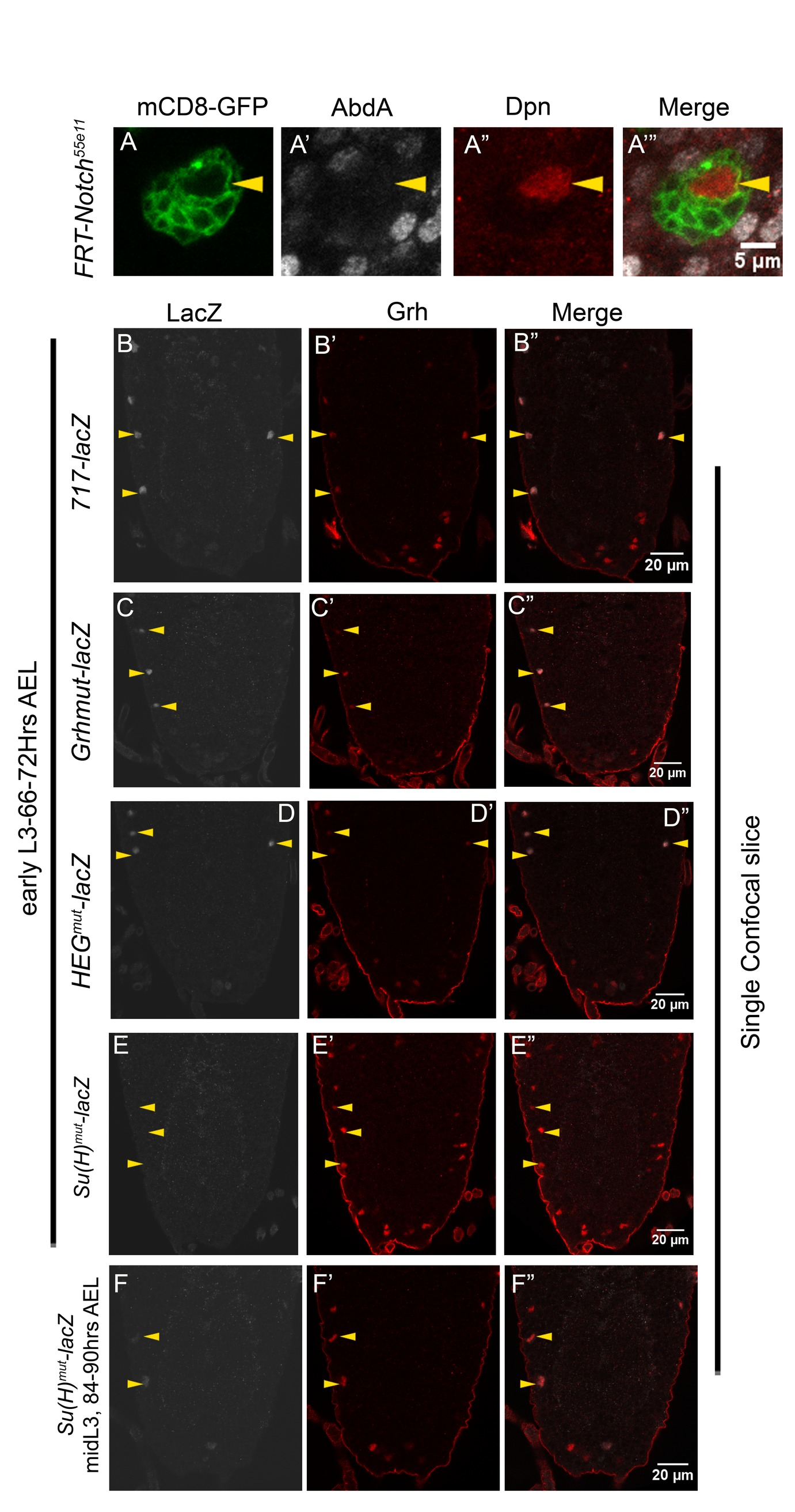

Supplement: S10 Fig — (A-A”‘) AbdA is down regulated in Notch55e11 clones in A1-A2 segments. (B-D) VNCs of 717-lacZ, 717-Grhmutant-lacZ, 717-HEGmutant-lacZ show normal expression in abdominal NBs in early L3 stage. (E-F) Abdominal pNBs of VNCs of 717-Su(H)mutant-lacZ do not express in early L3 stage (E), but the expression comes on in mid L3 stage (F). pNBs are indicated by yellow arrowheads. (TIF) [file pgen.1007043.s010.tif]
