# Supplementary material for: Combinatorial action of Grainyhead, Extradenticle and Notch in regulating Hox mediated apoptosis in Drosophila larval CNS
Source: PLoS Genet. 2017 Oct 12;13(10):e1007043. doi: 10.1371/journal.pgen.1007043 (PMC5667929; doi:10.1371/journal.pgen.1007043)
Supplement: S1 Text — Sequence of wild type and mutant binding motifs in 717bp enhancer. (DOCX) [file pgen.1007043.s011.docx]

**S1 Text**

***M22 deletion***

14.6 Kb genomic deletion called *M22* was generated by mobilizing MIMIC element inserted 9kb from 5’ region of *NBRR* (BDSC stock no. 30966). *M22* deletes entire *F3* fragment and 3kb overlapping region (including 1Kb *F3B3* and 717bp *F3B3B*, Fig-1A and supplementary Fig-2). We observed that heteroallelic combination of *M22* with *MM3* (a 54 Kb deletion including *NBRR* [[1](#_ENREF_1)]) also showed ectopic NBs in the abdominal region of CNS (Supp Fig-4B-C). We counted these ectopic NB in AbdA stained region (including A1-A2 segments) of larval CNS for wild type and various deletion combinations. In control VNCs, we found approximately 15 (14.3+/-3) pNBs (which were in abdominal segments A1-A2; Supp Fig 4A) and a much higher number of NB in the abdominal region of the larval VNCs for two heteroallelic combination *M22/MM3* (Supp Fig-4B-C, 161.9+/-12.7, n=20 VNCs) and *M22/H99* (Supp Fig-4C, 186.8+/-11.5, n=13 VNCs). These number were in agreement with *MM3/MM3* deletion as well (Supp fig-3D, 167.6+/-10.8, for n=12 VNCs). This analysis indicates that 14.6kb *M22* deletion uncovered the enhancer for abdominal pNB apoptosis.

The MiMIC element was mobilized in a Bloom Helicase mutant background (*BlmN1/D2* allelic combination, BDSC stock no. 28878 and 8657) to cause a deletion of flanking genomic sequences The extent of deletion was found to be 14.5kb as mapped using PCR. Details of the primers used are available on request.

**Identification of ectopic NBs in Dfd expressing region of SEG**

Dfd expressing region of SEG has 10 pNBs (5 NBs per hemisegment) in L2 stage (shown in green filled circle in Fig-4A). Four out of these10 pNBs undergo Dfd dependent apoptosis as animal progress from L2 to L3 stage of development [2]). The remaining six NB lineages (3 pNBs per hemisegment) are referred to as SA1, SA2 and SA3 (shown as hollow and filled red circles marked 1, 2, and 3 in Fig-4A). The relative positions of these 3 NB lineages within Dfd expressing region of SEG (Dfd-SEG) are relatively constant and are shown schematically in Fig-4A. Two of the three lineages (SA1 and SA2) are Dfd positive-they express Dfd in most of their progeny. The third lineage, i.e., SA3 expresses extremely low levels of Dfd (SA3) [[2](#_ENREF_2)].

In past, four pNB lineages which undergo Dfd dependent death have been identified by making MARCM clones mutant for Dfd (*Dfd^16^*) as well as for *Df(3L)-H99* clones and were classified as *Ect1^Dfd^* and *Ect2^Dfd^* lineages based on their position and axonal secondary tract projection [[2](#_ENREF_2)].

In our experiments, since we used *inscGAL4>UAS-mCD8-GFP* to drive the knockdown, all NB lineages in the region were marked with mCD8-GFP. Therefore, identification of ectopic lineages using axonal trajectory was informative but often had only limited value.

Hence, for the purpose of our experiments, we identified these four ectopic pNB lineages based on the following criteria: 1) Their specific location with respect to the 6 NBs which don’t die (SA1, SA2 and SA3), 2) Their position within Dfd expression domain in SEG and 3) whether the ectopic lineages also expressed Dfd. All these criteria are described below in detail.

First, we temporally blocked cell death through controlled expression of p35 in CNS using *inscGAL4;tub-gal80^ts^* from late embryonic or early L1 stage of development and dissected the specimen at late L3 stage of development. The two kind of temperature shift protocols followed are detailed in Sup Fig 8A-B. In both the cases, we could score 10 NB lineages (5 per hemisegment) in Dfd expressing region of SEG. The comparison of the p35 expressing VNCs and control VNCs clearly showed the relative location of 4 ectopic lineages in Dfd-SEG of CNS. One of the ectopic pNB lineages reproducibly appeared posterior to the SA2 and SA3 lineage towards the midline (which has been reported for the *ect^1^* Dfd lineage). The second lineage was always found laterally to the SA3 lineage and is thus probably the *ect^2^* Dfd lineage [[2](#_ENREF_2)].

Secondly, we observed that both the lineages were located near, but well within the posterior boundaries of the Dfd expressing cells. Also, we noticed that the progeny of both the lineages were always Dfd positive.

Based on these observations, we believe that the pNBs recovered in the p35 blocked state as well as in the knockdown background are the *Ect1^Dfd^* and *Ect2^Dfd^* lineages [[2](#_ENREF_2)]. These have been denoted as “e1” and “e2” in Fig-4A.

**Specific mutation of the motifs in 717bp enhancer.**

Grh and Su(H) binding sites are shown in bold. AT rich sites which are potential Hox and Exd sites are underlined as well. Mutations are shown in bold and small case for Grh and Su(H) sites. Mutation for AT rich sequences are shown in small case, bold and underlined as well.

| Motif-25 | CGGG**ATATC**AC**CAAACCATA**GAA**AATT**TGCGGTACA |
| --- | --- |
| *HEG^mutant^* | CGGGA**c**A**cg**AC**g**AA**cg**CA**gc**GAAA**cgTT**GCGcTACA |
| *Grh^mutant^* | CGGGATATCACCA**gcggg**TAGAAAATTTGCGGTACA |

| Motif -27 | **TATA**TGTGG**AACCA**CCTTGAAAGTC**TA**CTG**CCTGATTA**GT |
| --- | --- |
| *HEG^mutant^* | **cgcg**TGTGGAA**cg**ACgTTGAAAGTC**gc**CTGC**g**TGA**cg**AGT |
| *Grh^mutant^* | TATATGTGG**ggggg**CCTTGAAAGTCTACTGC**gccc**TTAGT |

| Motif -28 | AAGCCGAG**CCTGAATCAGGTA**CTCA**AATT**GTCGC |
| --- | --- |
| *HEG^mutant^* | AAGCCGAGC**g**TGAA**g**CA**c**GT**g**CTCAAA**g**TGTCGC |
| *Grh^mutant^* | AAGCCGAGC**gccc**A**gggc**GTACTCAAATTGTCGC |

| Motif -30 | GAA**ACAGGATAACCATAA**C**TAAT**CC |
| --- | --- |
| *HEG^mutant^* | GAAACA**c**GA**g**AAC**g**A**gg**ACT**gc**TCC |
| *Grh^mutant^* | GAAACA**c**GATAAC**g**ATAACTAATCC |

| Motif -31 | ACAC**AATT**TTC**TAA**G**AT**CC**ACTGGAAT**CGG**TAT**GCCG**ATA**GTT |
| --- | --- |
| *HEG^mutant^* | ACACA**cg**TTTC**g**AAGA**g**CCAC**g**GGA**c**TCGG**gcg**GCCG**gc**AGTT |
| *Grh^mutant^* | ACACAATTTTCTAAGATCC**ccg**GGAATCGGTATGCCGATAGTT |

| Motif -32 | TTGCATGTGCGG**ATATATATAT**GTGG**AACCA**CCTTG |
| --- | --- |
| *HEG^mutant^* | TTGCAcGTGCGG**gg**A**g**AT**cg**A**g**GTGGAA**cg**AC**g**TTG |
| *Grh^mutant^* | TTGCATGTGCGGATATATATATGTGG**ggcgg**CCTTG |

| Motif -33 | TACTG**CCTGATTA**GTG**TAAATAATT**G**CCAGT**CCTTG |
| --- | --- |
| *HEG^mutant^* | gcCTGC**g**TGA**cg**AGTG**cg**AA**gc**A**cg**GC**g**AGTCCTTG |
| *Grh^mutant^* | TACTGC**gccc**TTAGTGTAAATAATTGC**gccc**CCTTG |

| Motif -34 | GTTGC**CCAGT**G**CCTGATATTT**GTACTCC |
| --- | --- |
| *HEG^mutant^* | GTTGCCC**g**G**g**GCC**g**G**g**T**gc**TTGTgCTCC |
| *Grh^mutant^* | GTTGC**ggggg**G**ggggg**TATTTGTACTCC |

| Su(H)-1 | AAATTGACTTTG**TTGCGA**ATTTTATAGACC |
| --- | --- |
| *Su(H)-1^mutant^* | AAATTGACTTTGT**a**GC**c**AATTTTATAGACC |

| Su(H)-2&3 | ACCCAGGA**GAAAAC**C**CTTCGCA**GAACAAAAGACACAC |
| --- | --- |
| *Su(H)-2&3^mutant^* | ACCCAGGAGAA**gg**CCCTT**g**GC**t**GAACAAAAGACACAC |

| Su(H)-4 | AACATCCGA**GAAAAC**AAAAGATACAAAATG |
| --- | --- |
| *Su(H)-4^mutant^* | AACATCCGAGAA**gg**CAAAAGATACAAAATG |

| Su(H)-5 | ACTCAAATT**GTCGCAT**AGCGCGTCAAATTAAC |
| --- | --- |
| *Su(H)-5^mutant^* | ACTCAAATTGT**g**GC**t**TAGCGCGTCAAATTAAC |

| Su(H)-6 | CAGCGTAGA**ATGAGAA**TCCTTTTGCGC |
| --- | --- |
| *Su(H)-6^mutant^* | CAGCGTAGAA**a**GA**c**AATCCTTTTGCGC |

| Su(H)-7 | AAAATTGACATT**ATCGCAT**GACAAATGTAACAAG |
| --- | --- |
| *Su(H)-7^mutant^* | AAAATTGACATTAT**g**GC**t**TGACAAATGTAACAAG |

**References:**

1. Tan Y, Yamada-Mabuchi M, Arya R, St Pierre S, Tang W, et al. (2011) Coordinated expression of cell death genes regulates neuroblast apoptosis. Development 138: 2197-2206.

2. Kuert PA, Hartenstein V, Bello BC, Lovick JK, Reichert H (2014) Neuroblast lineage identification and lineage-specific Hox gene action during postembryonic development of the subesophageal ganglion in the Drosophila central brain. Dev Biol 390: 102-115.
