# Supplementary material for: Combinatorial action of Grainyhead, Extradenticle and Notch in regulating Hox mediated apoptosis in Drosophila larval CNS
Source: PLoS Genet. 2017 Oct 12;13(10):e1007043. doi: 10.1371/journal.pgen.1007043 (PMC5667929; doi:10.1371/journal.pgen.1007043)
Supplement: S1 Table — (DOCX) [file pgen.1007043.s012.docx]

Supplementary Table-1

|  | Size (kbs) | **Expression in NBs** | | |
| --- | --- | --- | --- | --- |
| ***Enh-lacZ lines*** |  | **Brain lobe** | **Thoracic** | **Abdominal** |
| *Frag-1* | 8kb | **+** | **++** | **-** |
| *Frag-2A* | 6kb | **+/-** | **-** | **-** |
| *Frag-2B* | 6kb | **+** | **+** | **-** |
| *Frag-3* | 8kb | **+** | **+** | **++** |
| *Frag-4* | 8kb | **+** | **+** | **+** |
| *F3B* | 4.5kb | **+** | **+** | **++** |
| *F3B3* | 1kb | **++** | **++** | **++** |
| *717* | 0.717Kb | **++** | **++** | **++** |
| **“++”Expressed Strongly; “+” Expressed;“+/-”Expressed weakly; “-”No expression** | | | | |
