# Supplementary material for: Combinatorial action of Grainyhead, Extradenticle and Notch in regulating Hox mediated apoptosis in Drosophila larval CNS
Source: PLoS Genet. 2017 Oct 12;13(10):e1007043. doi: 10.1371/journal.pgen.1007043 (PMC5667929; doi:10.1371/journal.pgen.1007043)
Supplement: S2 Table — (DOCX) [file pgen.1007043.s013.docx]

Supplementary Table-2

| Motif | **Grh** | **Exd** | **AbdA** | **AbdA-Exd** | **AbdA-Grh** | **Exd-Grh** | **AbdA-Exd-Grh** |
| --- | --- | --- | --- | --- | --- | --- | --- |
| **23** | **++** | **+/-** | **+/-** | **+/-** | **-** | **-** | **-** |
| **24** | **-** | **+/-** | **++** | **++** | **-** | **-** | **-** |
| **25** | **+** | **+/-** | **+/-** | **+** | **++** | **+/-** | **+/-** |
| **27** | **++** | **+/-** | **++** | **++** | **+** | **+/-** | **+** |
| **28** | **++** | **+/-** | **+** | **+** | **++** | **-** | **-** |
| **29** | **-** | **+/-** | **+** | **++** | **-** | **-** | **-** |
| **30** | **++** | **-** | **++** | **++** | **++** | **+/-** | **++** |
| **31** | **+/-** | **-** | **+/-** | **+** | **+/-** | **-** | **+/-** |
| **32** | **+** | **++** | **-** | **+** | **++** | **+/-** | **+** |
| **33** | **+** | **+** | **+** | **++** | **+/-** | **-** | **-** |

**“++”**represents good binding; “**+**”represents weak binding; “**+/-**”represents very weak binding; “**-**”represents no binding
